# Comparison of effect of CTG + STan with CTG alone on emergency Cesarean section rate: STan Australian Randomized controlled Trial (START)

**DOI:** 10.1002/uog.26279

**Published:** 2023-10-03

**Authors:** S. Kuah, B. Simpson, A. Salter, G. Matthews, J. Louise, J. Bednarz, E. Chandraharan, I. Symonds, A. McPhee, B. W. Mol, D. Turnbull, C. Wilkinson

**Affiliations:** ^1^ Women's and Children's Hospital North Adelaide SA Australia; ^2^ Women's and Children's Health, Adelaide Medical School University of Adelaide North Adelaide SA Australia; ^3^ School of Public Health, Faculty of Health and Medical Sciences University of Adelaide Adelaide SA Australia; ^4^ Women's and Children's Hospital, Faculty of Health and Medical Sciences University of Adelaide North Adelaide SA Australia; ^5^ South Australian Health and Medical Research Institute SAHMRI Women and Kids North Adelaide SA Australia; ^6^ Global Academy of Medical Education and Training Ltd London UK; ^7^ Adelaide Medical School, Faculty of Health and Medical Sciences University of Adelaide Adelaide SA Australia; ^8^ Department of Obstetrics and Gynaecology Monash University Clayton VIC Australia; ^9^ Aberdeen Centre for Women's Health Research, School of Medicine, Medical Sciences and Nutrition University of Aberdeen Aberdeen UK; ^10^ School of Psychology, Faculty of Health and Medical Sciences University of Adelaide Adelaide SA Australia

**Keywords:** cardiotocography, Cesarean section, CTG, fetal ECG, fetal electrocardiography, intrapartum fetal monitoring, randomized controlled trial, RCT, ST analysis, STan

## Abstract

**Objective:**

To investigate whether use of ST analysis of the fetal electrocardiogram (STan) as an adjunct to continuous cardiotocography (CTG) reduces the rate of emergency Cesarean section (EmCS) compared with CTG alone.

**Methods:**

This was a randomized controlled trial of patients with a singleton fetus in cephalic presentation at ≥ 36 weeks' gestation, requiring continuous electronic fetal monitoring during labor at a tertiary maternity hospital in Adelaide, Australia, between January 2018 and July 2021. Participants were randomized to undergo CTG + STan or CTG alone. The calculated sample size was 1818 participants. The primary outcome was EmCS. Secondary outcomes included metabolic acidosis, a composite adverse perinatal outcome, and other maternal and neonatal morbidity and safety outcomes.

**Results:**

The present study enrolled 970 women, of whom 967 were included in the primary analysis. EmCS occurred in 107/482 (22.2%) deliveries in the CTG + STan arm and in 107/485 (22.1%) in the CTG arm (adjusted relative risk, 1.02 (95% CI, 0.81–1.27); *P* = 0.89). There was no difference in the rate of adverse maternal or neonatal outcomes between arms.

**Conclusions:**

The addition of STan as an adjunct to continuous CTG did not reduce the EmCS rate. The smaller‐than‐anticipated sample size meant that this study was underpowered to detect absolute differences of ≤ 5% and, therefore, this negative finding could be due to a Type‐2 error. © 2023 The Authors. *Ultrasound in Obstetrics & Gynecology* published by John Wiley & Sons Ltd on behalf of International Society of Ultrasound in Obstetrics and Gynecology.

**CONTRIBUTION**

*What are the novel findings of this work?*

This comprehensive randomized controlled trial is the first Australian experience with ST analysis of the fetal electrocardiogram (STan) as an adjunct to continuous cardiotocography (CTG) during the intrapartum period. We found that the addition of STan did not reduce our institution's high and entrenched emergency Cesarean section (EmCS) rate, although our outcomes may have been subject to Type‐2 error due to a smaller‐than‐anticipated sample size. However, this trial has the advantage of not being confounded by use of fetal (scalp) blood sampling in both arms as similar European studies may have been.

*What are the clinical implications of this work?*

Although there was no evidence that STan as an adjunct to CTG reduced the rate of EmCS in women requiring continuous electronic fetal monitoring in labor, this work demonstrates that STan may be introduced safely to a large tertiary maternity unit and may provide clinical benefit with respect to neonatal outcome, as seen with the direction of effect of our data.

## INTRODUCTION

The Cesarean section (CS) rate in Australia continues to rise and, standing at 33.7%, remains higher than the Organization for Economic Co‐operation and Development (OECD) average of 28.1%^1^. This organization ranked Australia as having the 8^th^ highest CS rate^1^ of 34 OECD countries in 2017. Continuous cardiotocography (CTG), used widely for monitoring intrapartum fetal wellbeing, is associated with a reduced rate of neonatal seizures but an increased rate of operative delivery^2^. The high false‐positive rate of CTG may be contributing to the rising emergency CS (EmCS) rate by falsely identifying fetuses as being compromised in labor^3^.

Several methods have been proposed as adjuncts to CTG to reduce unnecessary EmCS for suspected fetal hypoxia/acidosis indicated by abnormal/pathological CTG patterns^4^, ^5^. One such approach is CTG incorporating ST analysis of the fetal electrocardiogram (STan; Neoventa Medical AB, Mölndal, Sweden)^6^. STan assists with detection of hypoxic stress by identifying changes to the ST segment of the fetal electrocardiogram^7^. This may reduce false‐positive diagnosis of fetal hypoxia/acidosis, better identify hypoxic stress and allow for timely intervention, including intrauterine resuscitation^5^, ^8^ and delivery.

STan as an adjunct to CTG (CTG + STan) was introduced to the Women's and Children's Hospital (WCH), Adelaide, Australia, in 2015, as a standard of care alongside the traditional standard of care of CTG alone. To our knowledge, this was the first utilization of CTG + STan in an Australian setting. To date, CTG + STan has been examined in eight randomized controlled trials (RCTs)^9^, ^10^, ^11^, ^12^, ^13^, ^14^, ^15^, ^16^. The three most recent meta‐analyses concluded that CTG + STan confers minimal or no benefit with regards to operative delivery rate or perinatal outcome^17^, ^18^, ^19^. Notably, RCTs evaluating STan vary in the choice of primary outcome, maternal and fetal risk status, labor management protocol, obstetric practice and STan guidelines for the management of ST events.

A pilot study of 162 women^20^ motivated our published study protocol^21^. We hypothesized that utilizing CTG + STan in women at ≥ 36 weeks' gestation with a singleton fetus in cephalic presentation, and requiring continuous electronic fetal monitoring (CEFM) during labor as per Royal Australian and New Zealand College of Obstetricians and Gynaecologists (RANZCOG) guidelines^5^, would result in a clinically meaningful reduction in the proportion of women undergoing EmCS, from 17% to 12% or lower, compared with CTG alone.

## METHODS

### Trial oversight

The STan Australian Randomized controlled Trial (START) was a single‐center, parallel‐group RCT conducted at a tertiary‐level facility (WCH, Adelaide, Australia). The study protocol has been published previously^21^. The trial steering committee (S.K., B.S., A.S., A.M. and C.W.) provided oversight for the trial. An external independent Data Safety Monitoring Committee (DSMC; members listed in Appendix S1) monitored adverse events, compliance with trial protocol and progress of recruitment. The DSMC was blinded to the type of monitoring received and met virtually to consider reports and ratify the investigators' opinions on whether events were related to the assigned intrapartum fetal monitoring method. The committee had the power to stop or modify the study. This study was approved prior to commencement by the Women's and Children's Health Network Human Research Ethics Committee (HREC/17/WCHN/14) and was registered prospectively with the Australian and New Zealand Clinical Trials Registry (ACTRN1261800006268).

### Participants

Women aged ≥ 18 years with a singleton fetus in cephalic presentation, who were able to give informed consent, were literate in English and did not meet any of the exclusion criteria, were eligible to participate. Exclusion criteria were: pregnancy < 36 weeks of gestation; planned CS delivery; placenta previa or vasa previa requiring CS; contraindication for use of fetal scalp electrode (FSE); previous participation in START; no clinical indication for CEFM; or known fetal structural or functional cardiac anomaly. Clinical indications for CEFM were based on antenatal or intrapartum factors that increase the risk of fetal compromise, as per RANZCOG guidelines (Table S1)^5^. Written informed consent was obtained prior to onset of labor, in early labor or after epidural analgesia.

### Trial procedures

Eligible consenting women were randomized once amniotic membranes were naturally or artificially ruptured and it was expected that labor was going to establish and progress, or during established labor up until active second stage of labor. Participants were randomized to either CTG + STan (intervention group) or CTG alone (control group) in a 1:1 ratio, stratified for parity (0 *vs* ≥ 1). The computer‐generated randomization schedule was prepared by an independent statistician who was not otherwise involved with the trial. Allocation utilized a telephone‐based system provided by the National Health and Medical Research Council Clinical Trials Centre at the University of Sydney, Sydney, Australia. This was a pragmatic trial, as it was not possible to blind participants and staff providing care to the assigned arm of the trial; however, the statistician performing the analysis was blinded to the identity of the study arms. Detailed descriptions of study arms were published in the trial protocol^21^ and are available in Appendix S2. The decision for and timing of EmCS or operative vaginal delivery lay with the delivery suite consultant or private obstetrician, guided by STan guidelines (Figure S1)^22^ and/or RANZCOG guidelines^5^, with additional consideration of the individual clinical situation and concurrent demands within the delivery suite. Clinical observation and data collection commenced at randomization, and data were collected for mothers and infants until 6 weeks after birth.

### Outcomes

The primary outcome was EmCS (yes/no). Secondary outcomes, prespecified in the published protocol^21^ and detailed in the statistical analysis plan (SAP; Appendix S3), were classified into delivery, maternal, neonatal and safety outcomes. Delivery outcomes included: delivery method (spontaneous vaginal, EmCS, forceps or vacuum‐assisted); episiotomy with spontaneous vaginal delivery; EmCS for fetal distress; instrumental delivery for fetal distress; and operative delivery in second stage of labor. Maternal outcomes included: complications of labor and delivery (meconium‐stained amniotic fluid, chorioamnionitis, postpartum hemorrhage, shoulder dystocia); oxytocin infusion for labor augmentation; epidural use; length of labor after randomization; length of second stage of labor; length of hospital stay; and readmission within 6 weeks after birth. Neonatal outcomes included: metabolic acidosis indicated by cord gases; respiratory distress at delivery; 5‐min Apgar score; 5‐min Apgar score ≤ 6; 5‐min Apgar score ≤ 3; neonatal seizure(s); use of Brainz monitoring (Natus Medical Inc., Newington, NSW, Australia); neonatal encephalopathy; hypoxic ischemic encephalopathy; requirement for cooling; proven infection; antibiotic use; composite adverse perinatal outcome (defined as any of the following: infant death (intrapartum or neonatal), 5‐min Apgar score ≤ 3, neonatal seizure, umbilical artery blood pH ≤ 7.05 and base excess in extracellular fluid (BE_ecf_) ≤ −12.0 mmol/L, intubation for ventilation at delivery or presence of hypoxic ischemic encephalopathy); fetal blood sampling (FBS); FSE use; complications from use of FSE; highest level of neonatal admission and length of stay; jaundice requiring phototherapy; meconium aspiration syndrome; major congenital malformation; and readmission within 6 weeks after birth. Safety outcomes included: maternal death; maternal intensive care unit admission; intrapartum fetal death; and neonatal death.

### Sample size calculation

European RCTs^10^, ^11^, ^12^, ^13^ comparing CTG + STan with CTG alone in countries that have low EmCS rates relative to our Australian institution have demonstrated no further reduction in EmCS rate. However, meta‐analyses have shown statistically significant reductions in FBS^19^, ^23^, which is utilized rarely in the trial institution. The relevance of this to our sample size calculation is that indications for FBS in these countries are likely to be the same as those for EmCS for suspected fetal distress in the trial institution. We believe that reduced usage of FBS observed in the European trials of STan may manifest as reduced EmCS if CTG + STan is used appropriately in our institution.

Therefore, sample size was based on data from our pilot study^20^, FBS reduction rates and the observed rate of EmCS at the WCH. The proportion of EmCS deliveries in the CTG only (control) group was expected to be approximately 17%, and the sample size was intended to provide 80% power (with two‐sided α = 0.05) to detect a 5% absolute reduction in the rate of EmCS in the CTG + STan group (i.e. from 17% to 12%). Allowing for 10% dropout rate after consenting to participate, a further 22% attrition rate due to lack of clinical indication for fetal monitoring and a further 5% non‐compliance rate in the CTG + STan group, it was estimated that 2588 women would need to consent, to allow for at least 1818 women (909 per group) to be randomized.

### Statistical analysis

Analysis of data was performed by an independent statistician (J.B.), blinded to group assignment, at Adelaide Health Technology Assessment (AHTA), School of Public Health, University of Adelaide, Adelaide, Australia, using Stata version 15 (StataCorp., College Station, TX, USA). Analysis followed the prespecified SAP (Appendix S3) and was performed using an intention‐to‐treat approach, whereby participants were analyzed according to the group to which they were randomized. A secondary per‐protocol sensitivity analysis, including only participants for whom fetal monitoring was undertaken according to study protocol, was performed for the primary outcome only. An interim analysis was planned for reporting to the DSMC after the recruitment of 800 women; however, this did not occur due to delays and slower recruitment during the COVID‐19 pandemic resulting in reduced sample size.

For dichotomous outcomes, including the primary outcome of EmCS (yes/no), log‐binomial regression was used to estimate the relative risk of the outcome, with 95% CI, in the CTG + STan group compared with the CTG group. Log‐Poisson regression with robust variance was used if the log‐binomial regression failed to converge. For continuous outcomes, linear regression was used to estimate the mean difference and 95% CI between groups. Ordinal outcomes were analyzed using ordinal logistic regression and survival outcomes were analyzed using parametric survival models. Both unadjusted and adjusted analyses were performed, with the adjusted analysis considered primary. All adjusted analyses included parity (as a stratification variable) along with additional covariates prespecified as related to the relevant outcome, including maternal body mass index (BMI), private‐patient status, maternal age (< 35 years *vs* ≥ 35 years), previous CS delivery, higher risk pregnancy/delivery and induction of labor. Full details of all analysis models may be found in the SAP (Appendix S3). To explore potential heterogeneity of treatment effects, preplanned subgroup analyses were conducted for the primary outcome within subgroups defined by selected baseline characteristics, including parity, BMI category, private‐patient status, maternal age ≥ 35 years, previous delivery by CS, higher risk pregnancy/delivery and induction of labor. Note that 95% CIs were not adjusted for multiplicity and, as such, may not be used in place of hypothesis testing. Data were complete, with the exception of the secondary outcome, infant 5‐min Apgar score (0.1% missing) and maternal BMI at baseline (2.2% missing). Therefore, complete case analyses were performed for all outcomes, in accordance with the strategies for handling missing data outlined in the SAP. Continuous data were presented as mean ± SD, unless distribution of the variable was skewed, in which case median (interquartile range) was reported; categorical variables were presented as *n* (%). *P*‐values < 0.05 were considered significant.

## RESULTS

### Recruitment and characteristics of participants

**Figure 1:**
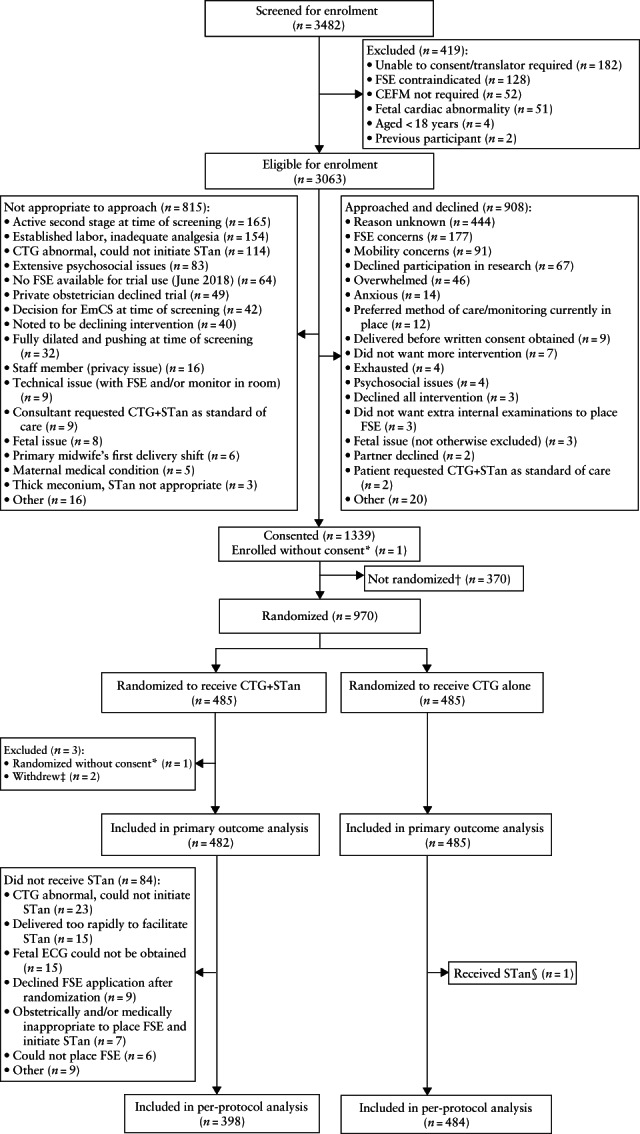
Flowchart summarizing screening, enrolment, randomization and analysis in STan Australian Randomized controlled Trial (START). *One woman was randomized mistakenly before consent was obtained and, when approached, declined to participate, as did not want fetal scalp electrode (FSE). †Reasons listed in Tables S3 and S4. ‡Both women verbally requested withdrawal from study after randomization to cardiotocography (CTG) plus ST analysis (STan), as changed mind and did not want FSE. §One participant allocated to CTG received STan monitoring in addition when ST analysis started automatically after FSE was applied for clinical reasons; no STan events occurred. CEFM, continuous electronic fetal monitoring; ECG, electrocardiogram; EmCS, emergency Cesarean section.

Recruitment to the trial began on 22 January 2018 and ended on 29 July 2021 due to fund depletion, allowing for completion of data collection and analysis prior to the end of the funding agreement in December 2021. Consent was obtained from 1339 women and most consents were obtained either prior to labor (497/1339 (37.1%)) or in early labor (510/1339 (38.1%)) (Table S2). Of the planned sample size of 1818 participants, a total of 970 (53.4%) were recruited during the 3.5‐year recruitment period and were assigned randomly to either CTG + STan (*n* = 485) or CTG alone (*n* = 485) (Figure 1, Tables S3 and S4). Three women in the CTG + STan group were excluded prior to analysis. The baseline characteristics of the two groups were similar (Tables 1 and S5).

**Table 1:** Baseline characteristics of study population, according to whether they were randomized to receive cardiotocography (CTG) with ST analysis (STan) or CTG alone

Characteristic	CTG + STan (*n* = 482)	CTG (*n* = 485)	Total (*n* = 967)
Maternal age (years)	30.8 ± 5.2	30.9 ± 5.1	30.9 ± 5.1
GA at randomization (weeks)	39.1 ± 1.2	39.1 ± 1.1	39.1 ± 1.2
Cervical dilation at randomization (cm)	3.0 (3.0–5.0)	3.0 (3.0–5.0)	3.0 (3.0–5.0)
Maternal early‐pregnancy BMI*	27.6 (23.4–34.7)	26.5 (23.1–33.1)	27.1 (23.3–33.7)
< 30.0 kg/m^2^	294 (62.3)	313 (66.0)	607 (64.2)
30.0–34.9 kg/m^2^	65 (13.8)	64 (13.5)	129 (13.6)
35.0–39.9 kg/m^2^	65 (13.8)	48 (10.1)	113 (11.9)
≥ 40.0 kg/m^2^	48 (10.2)	49 (10.3)	97 (10.3)
Private patient	55 (11.4)	54 (11.1)	109 (11.3)
Nulliparous	289 (60.0)	290 (59.8)	579 (59.9)
Previous Cesarean section	19 (3.9)	30 (6.2)	49 (5.1)
Induction of labor†	400 (83.0)	384 (79.2)	784 (81.1)
Nulliparous	223/400 (55.8)	213/384 (55.5)	436/784 (55.6)
Oxytocin augmentation prior to randomization	296 (61.4)	284 (58.6)	580 (60.0)
Epidural analgesia prior to randomization	229 (47.5)	236 (48.7)	465 (48.1)
Higher risk pregnancy/delivery			
Type‐1/Type‐2/gestational diabetes mellitus	120 (24.9)	127 (26.2)	247 (25.5)
Hypertension‡	46 (9.5)	52 (10.7)	98 (10.1)
Pre‐eclampsia	16 (3.3)	15 (3.1)	31 (3.2)
Intrauterine growth restriction	39 (8.1)	24 (4.9)	63 (6.5)
PPROM	4 (0.8)	1 (0.2)	5 (0.5)
Obstetric cholestasis	8 (1.7)	7 (1.4)	15 (1.6)
Indications for CEFM§	3.7 ± 1.2	3.6 ± 1.2	3.7 ± 1.2
Birth weight	3307.3 ± 472.1	3344.4 ± 470.1	3325.9 ± 471.2
≤ 2500 g	28 (5.8)	20 (4.1)	48 (5.0)
≥ 4000 g	35 (7.3)	39 (8.0)	74 (7.7)
Male infant sex	248 (51.5)	246 (50.7)	494 (51.1)

Data are given as mean ± SD, median (interquartile range), *n* (%) or *n*/*N* (%).

* Data reported for 472 patients in CTG + STan group, 474 in CTG group and 946 overall.

† Reason for induction of labor listed in Table S5.

‡ Pre‐existing or gestational.

§ Continuous electronic fetal monitoring (CEFM) was according to Royal Australian and New Zealand College of Obstetricians and Gynaecologists (RANZCOG) recommendations^5^ and reasons (iatrogenic, medical, obstetric) for CEFM are listed in Table S5.

BMI, body mass index; GA, gestational age; PPROM, preterm prelabor rupture of membranes.

### Outcomes

The rate of EmCS in the CTG + STan (22.2%) and CTG (22.1%) arms was not significantly different (adjusted relative risk, 1.02 (95% CI, 0.81–1.27); *P* = 0.89) (Table 2). Similar results were found for the secondary per‐protocol analysis (Table S6). Prespecified subgroup analyses were performed for the primary outcome and no differential treatment effects (i.e. modification) were found (Table S7).

**Table 2:** Primary outcome and secondary delivery and maternal outcomes for study population, according to whether they were randomized to receive cardiotocography (CTG) with ST analysis (STan) or CTG alone

Outcome	CTG + STan (*n* = 482)	CTG (*n* = 485)	Adjusted* RR (95% CI)	*P*
Delivery method					
EmCS (primary outcome)	107 (22.2)	107 (22.1)	1.02 (0.81–1.27)	0.89
Spontaneous	258 (53.5)	265 (54.6)	0.96 (0.86–1.07)	0.44
With episiotomy†	61/258 (23.6)	51/265 (19.2)	—	—
Forceps†	79 (16.4)	85 (17.5)	—	—
Vacuum‐assisted†	38 (7.9)	28 (5.8)	—	—
Indication					
EmCS				
Suspected fetal distress	40/107 (37.4)	52/107 (48.6)	0.81 (0.55–1.20)	0.29
Dystocia‡	65/107 (60.7)	53/107 (49.5)	1.29 (0.93–1.78)	0.15
Other†	2/107 (1.9)	2/107 (1.9)	—	—
Forceps or vacuum‐assisted					
Suspected fetal distress	82/117 (70.1)	67/113 (59.3)	1.25 (0.93–1.68)	0.14
Dystocia‡	29/117 (24.8)	40/113 (35.4)	0.75 (0.48–1.19)	0.22
Other†	6/117 (5.1)	6/113 (5.3)	—	—
Operative delivery§ in second stage of labor¶	132/389 (33.9)	131/396 (33.1)	1.03 (0.86–1.23)	0.78
Oxytocin augmentation commenced after randomization	142 (29.5)	137 (28.2)	1.04 (0.85–1.26)	0.72
Epidural use after randomization	176 (36.5)	176 (36.3)	1.01 (0.85–1.19)	0.94

Data are given as *n* (%) or *n*/*N* (%), unless stated otherwise.

* Relative risk (RR) of outcome (CTG + STan *vs* CTG) derived from log‐binomial regression model.

Due to convergence issues, adjusted treatment effect estimate derived from log‐Poisson regression model with robust variance for delivery method.

Spontaneous delivery, indication for emergency Cesarean section (EmCS), indication for forceps or vacuum‐assisted delivery and operative delivery in second stage of labor adjusted for parity (0 *vs* ≥ 1), maternal body mass index (BMI) at baseline and previous Cesarean delivery.

EmCS adjusted for parity (0 *vs* ≥ 1), maternal BMI at baseline, private‐patient status, maternal age ≥ 35 years, previous Cesarean delivery, higher risk pregnancy/delivery and induction of labor.

Oxytocin augmentation and epidural analgesia adjusted only for parity (0 *vs* ≥ 1).

† Not analyzed as per statistical analysis plan (Appendix S3).

‡ *Post‐hoc* analysis adjusted for parity (0 *vs* ≥ 1), maternal BMI at baseline, private‐patient status, maternal age ≥ 35 years, previous Cesarean delivery, higher risk pregnancy/delivery and induction of labor.

§ Forceps, vacuum‐assisted or EmCS.

¶ Outcome defined only for subset of women who reached second stage of labor.

We found no difference in the proportion of spontaneous deliveries between trial arms (Table 2). Similarly, there was no difference between arms in the proportions of EmCS or instrumental (forceps or vacuum‐assisted) deliveries that were indicated for suspected fetal distress. Likewise, *post‐hoc* analysis showed that the proportions of EmCS and instrumental delivery indicated for labor dystocia were similar between groups. We found no difference in the rate of operative delivery between participants in each arm who reached the second stage of labor (Table 2), including on sensitivity analysis (Table S8). Length of labor after randomization was similar between arms (Table S9).

Maternal postrandomization characteristics (including complications of labor and delivery, length of hospital stay and rate of readmission) and safety outcomes were similar between arms (Table S10). Neonatal characteristics and safety outcomes were also similar between groups (Table S11). FBS was used only once, and occurred in the CTG arm (1/485 (0.2%)) (Table S11). FSEs were utilized for fetal monitoring in 451/482 (93.6%) of the CTG + STan arm and 355/485 (73.2%) of the CTG arm, and complications from FSE usage occurred at a similar rate in both arms (Table S12).

Valid paired umbilical cord gas measurements were obtained for 841/967 (87.0%) neonates. To be valid, the arterial pH had to be lower than the venous pH by ≥ 0.03 units^24^. Umbilical cord arterial pH ≤ 7.05 and BE_ecf_ ≤ −12.0 mmol/L occurred in 3/424 (0.7%) neonates in the CTG + STan arm and 4/417 (1.0%) in the CTG arm (Table 3). Neonatal metabolic acidosis, defined^25^ using the current consensus threshold of arterial cord blood pH < 7.00, occurred in no neonates in the CTG + STan arm and in 2/417 (0.5%) neonates in the CTG arm. We found no difference in frequency of respiratory distress (Table 3). 5‐min Apgar score ≤ 6 was reported in 2/481 (0.4%) and 5/485 (1.0%) neonates in the CTG + STan and CTG arms, respectively. We found no difference in continuous infant 5‐min Apgar score (Table S13). The rate of composite adverse perinatal outcome was not analyzed due to insufficient events (Table 3). We found no difference in the rate of neonatal complications, namely jaundice requiring phototherapy and antibiotic use, although the rates of meconium aspiration syndrome and confirmed infection were not analyzed due to insufficient events.

**Table 3:** Secondary neonatal outcomes for study population, according to whether they were randomized to receive cardiotocography (CTG) with ST analysis (STan) or CTG alone

Outcome	CTG + STan (*n* = 482)	CTG (*n* = 485)	Adjusted* RR (95% CI)	*P*
Metabolic acidosis†				
UA pH ≤ 7.05 and BE_ecf_ ≤ −12.0 mmol/L‡	3/424 (0.7)	4/417 (1.0)	—	—
UA pH < 7.00 and BE_ecf_ ≤ −12.0 mmol/L‡	0/424 (0)	2/417 (0.5)	—	—
Respiratory distress§	57 (11.8)	69 (14.2)	0.84 (0.60–1.16)	0.28
5‐min Apgar ≤ 6‡	2/481 (0.4)††	5 (1.0)	—	—
5‐min Apgar ≤ 3‡	0 (0)	0 (0)	—	—
Composite adverse perinatal outcome‡, ¶	6 (1.2)	4 (0.8)	—	—
Neonatal complication				
Jaundice requiring phototherapy	59 (12.2)	70 (14.4)	0.85 (0.61–1.17)	0.31
Meconium aspiration syndrome‡	2 (0.4)	1 (0.2)	—	—
Use of antibiotics	72 (14.9)	75 (15.5)	0.96 (0.72–1.29)	0.80
Confirmed infection‡	3 (0.6)	1 (0.2)	—	—
Neonatal admission**	133 (27.6)	143 (29.5)	0.94 (0.77–1.14)	0.53
Neonatal readmission**	38 (7.9)	50 (10.3)	0.77 (0.51–1.14)	0.19

Data are given as *n*/*N* (%) or *n* (%), unless stated otherwise.

* Relative risk (RR) of outcome (CTG + STan *vs* CTG) derived from log‐binomial regression model, adjusted for parity (0 *vs* ≥ 1).

† Outcome defined only for neonates with valid paired cord blood gases.

‡ Not analyzed due to no or insufficient number of events.

§ Defined as requirement for continuous positive airway pressure, intermittent positive pressure ventilation or intubation at delivery.

¶ Defined as any of the following: infant death (intrapartum or neonatal), 5‐min Apgar score ≤ 3, neonatal seizure, umbilical artery (UA) blood pH ≤ 7.05 and base excess in extracellular fluid (BE_ecf_) ≤ −12.0 mmol/L, intubation for ventilation at delivery or presence of hypoxic ischemic encephalopathy.

** To neonatal intensive care unit (NICU), special care baby unit (SCBU) or postnatal ward.

In study institution, NICU is where multidisciplinary team cares for babies who require respiratory support (including intubation), central line management and continuous observation; SCBU cares for babies needing short‐ and long‐term observation, respiratory support other than intubation and specialized care; postnatal ward is for admitted patients requiring, for example, intravenous antibiotics or phototherapy for jaundice, but infant remains with mother.

†† Data missing for one patient.

Neonatal admission and readmission rates are reported in Table 3. Neonatal admission and readmission were described with respect to the highest of four levels of care (none, postnatal ward, special baby care unit, neonatal intensive care unit) received by the infant. We found no difference in the odds of admission to higher levels of care (*vs* lower levels of care) for the CTG + STan group relative to the CTG group, for either of neonatal admission or neonatal readmission (Table S14). Infant length of stay is reported in Table S15, for which we found no difference between arms.

### Severe adverse events

The DSMC raised no concerns. Table S16 details the severe adverse event reports (10 in CTG + STan arm and 15 in CTG arm) that were documented during the trial. The rate and type of event were similar between arms.

### STan protocol adherence

All STan traces were audited (by B.S. and G.M.) with respect to initiation, quality and expedited delivery recommendations as per STan guidelines^22^ and results are reported in Appendix S4.

## DISCUSSION

There was no difference in the proportion of pregnancies delivered by EmCS between the CTG + STan and CTG groups. However, we acknowledge that this study was underpowered to detect absolute differences of ≤ 5% and, therefore, this finding could be due to a Type‐2 error, i.e. a difference may exist, but we were underpowered to detect it. The number of women actually randomized to the study was 970 (485 per group), which was 53% (970/1818) of the planned sample size. With the actual number randomized, the statistical power to detect the original difference of 5% was only approximately 56%. At the time of performing power and sample size calculations for the trial^20^, the rate of EmCS at the trial institution was approximately 18% (WCH Clinical Information Service (CIS) data, 2015); this proportion was revised down to 17% for the power calculation, due to introduction of a rigorous fetal surveillance education program mandated by the health department for all obstetric and midwifery staff involved in intrapartum care. We considered that this program itself would decrease the WCH EmCS rate, regardless of the addition of STan as an adjunct to CTG.

The overall EmCS rate in the study cohort of 22.1% was higher compared with the institutional EmCS rate of 20% (WCH CIS data, 2018–2021; includes trial participants) over the trial period. We believe that the higher rate observed in the trial may be due to higher participant acuity: women who did not require CEFM, and were therefore lower risk, were excluded from the study, while women undergoing induction of labor were over‐represented in the study (81.1% compared with the averaged institutional rate of 47.9%; WCH CIS data, 2018–2021; includes trial participants). We consider that our trial population was not low risk, and that it shares similarities with the participants recruited to the RCTs conducted in The Netherlands^12^ and Sweden^13^, of which both had a lower EmCS rate in the CTG arm (13.8% and 9.1%, respectively). Just prior to commencing this study, the EmCS rate was 19.8% in the total WCH population, which included also low‐risk women, i.e. women in whom intermittent auscultation was utilized during labor. Therefore, an opportunity (recognized by the funding body) existed to ascertain whether STan, as an adjunct to CTG, would lower the EmCS rate in an environment of high CS usage.

The research team's commitment to prioritizing the gold‐standard RCT design^26^ was supported with a sustained and commensurately resourced effort to tackle the well‐described challenges of intrapartum consent^27^, ^28^. COVID‐19 pandemic restrictions compounded recruitment issues, with limitations on non‐critical activity in public health services mandated from March 2020. However, barriers to recruitment predated the pandemic: there was reluctance among midwives to recruit participants, and among women to participate, due to the perceived invasiveness of FSE and the impairment of mobility associated with STan, resulting from its incompatibility with telemetry. Post‐trial audit of recruitment confirmed that concerns about FSE use and/or mobility reduction were the primary reason for 30% of women declining participation (Figure 1). Acknowledging this reluctance for women to consent due to FSE and/or mobility concerns, provisional consent was offered when extra recruitment staff were available (from September 2020 to June 2021) to streamline the consenting process. Women would provide written informed consent, under the proviso that they would undergo randomization only if a FSE was required for routine clinical care outside of the trial and/or mobility concerns were voided due to initiation of epidural analgesia. Although this increased consent rates, compared with full consents (i.e. not provisional on the clinical use of FSE and/or epidural analgesia), of which 91% resulted in enrolment, only 28% of women consenting provisionally to participate in the clinical trial were enrolled.

The main strength of this study is its being the first RCT on STan as an adjunct to CTG in a region with a relatively high CS rate. Unlike the centers in which European RCTs were conducted, our institution does not have a high rate of FBS and, therefore, this study could compare the true effect of STan without the confounding effect of FBS on both arms. The main limitation of this study was that we could not achieve the necessary rate of recruitment, primarily due to the reluctance of women and midwives to utilize STan, given its requirement for FSE and incompatibility with telemetry, and therefore this study was underpowered.

In this trial, which was underpowered to detect a clinically meaningful difference for the primary outcome of EmCS, there was no evidence that STan as an adjunct to CTG reduced the rate of EmCS in women requiring CEFM in labor. Similarly, while the observed rates of poor neonatal outcome were mostly lower in the CTG + STan arm, these differences were not statistically significant and, further, there was no difference in the rate of metabolic acidosis. Therefore, we cannot conclusively determine whether STan is an effective adjunct to CTG for intrapartum fetal monitoring.

## Supporting information


**Appendix S1** Summary of Data Safety Monitoring Committee (DSMC)
**Appendix S2** Description of study arms
**Appendix S3** START trial statistical analysis plan
**Appendix S4** ST analysis (STan) protocol adherence
**Table S1** Clinical indications for continuous electronic fetal monitoring (CEFM) as per Royal Australian and New Zealand College of Obstetricians and Gynaecologists (RANZCOG) clinical guideline
**Table S2** Labor progress at time of consent
**Table S3** Reasons for consents not resulting in randomization
**Table S4** Reasons for provisional consents not resulting in randomization
**Table S5** Reasons for induction of labor and continuous electronic fetal monitoring
**Table S6** Secondary per‐protocol analysis for primary outcome
**Table S7** Secondary subgroup analyses for primary outcome
**Table S8** Sensitivity analysis for operative delivery in second stage of labor
**Table S9** Secondary maternal outcomes: length of labor after randomization and length of second stage of labor
**Table S10** Maternal postrandomization characteristics
**Table S11** Neonatal postrandomization characteristics
**Table S12** Postrandomization characteristics: fetal heart‐rate‐monitoring method and associated complications
**Table S13** Continuous secondary neonatal outcome: 5‐min Apgar score
**Table S14** Ordinal secondary neonatal outcomes: level of neonatal admission and readmission
**Table S15** Continuous secondary neonatal outcome: infant length of stay
**Table S16** Data Safety Monitoring Committee (DSMC) severe adverse event reports
**Figure S1** Fetal heart rate classification system for cardiotocography plus ST analysis and corresponding management guidelines.

## Data Availability

Author elects to not share data
