# Supplementary material for: Comparison of effect of CTG + STan with CTG alone on emergency Cesarean section rate: STan Australian Randomized controlled Trial (START)
Source: Ultrasound Obstet Gynecol. 2023 Oct 3;62(4):462–70. doi: 10.1002/uog.26279 (PMC10946943; doi:10.1002/uog.26279)
Supplement: Supplementary file 1 — Appendix S1 Summary of Data Safety Monitoring Committee (DSMC) Appendix S2 Description of study arms Appendix S3 START trial statistical analysis plan Appendix S4 ST analysis (STan) protocol adherence Table S1 Clinical indications for continuous electronic fetal monitoring (CEFM) as per Royal Australian and New Zealand College of Obstetricians and Gynaecologists (RANZCOG) clinical guideline Table S2 Labor progress at time of consent Table S3 Reasons for consents not resulting in randomization Table S4 Reasons for provisional consents not resulting in randomization Table S5 Reasons for induction of labor and continuous electronic fetal monitoring Table S6 Secondary per‐protocol analysis for primary outcome Table S7 Secondary subgroup analyses for primary outcome Table S8 Sensitivity analysis for operative delivery in second stage of labor Table S9 Secondary maternal outcomes: length of labor after randomization and length of second stage of labor Table S10 Maternal postrandomization characteristics Table S11 Neonatal postrandomization characteristics Table S12 Postrandomization characteristics: fetal heart‐rate‐monitoring method and associated complications Table S13 Continuous secondary neonatal outcome: 5‐min Apgar score Table S14 Ordinal secondary neonatal outcomes: level of neonatal admission and readmission Table S15 Continuous secondary neonatal outcome: infant length of stay Table S16 Data Safety Monitoring Committee (DSMC) severe adverse event reports Figure S1 Fetal heart rate classification system for cardiotocography plus ST analysis and corresponding management guidelines. [file UOG-62-462-s001.docx]

**Supplementary Material**

This appendix has been provided by the authors to give readers
additional information about their work.

Supplement to:

**Comparing the effect of CTG+STan with CTG alone on emergency Cesarean section rate: STan Australian Randomized controlled Trial (START)**

**Table of Contents**

[1. Appendix S1. Summary of Data Safety Monitoring Committee (DSMC) 3](#_Toc136420198)

[2. Table S1. Clinical indications for continuous electronic fetal monitoring (CEFM) as per Royal Australian and New Zealand College of Obstetricians and Gynaecologists (RANZCOG) clinical guideline^1^ 4](#_Toc136420199)

[3. Appendix S2. Description of study arms 5](#_Toc136420200)

4. Figure S1. Fetal heart rate (FHR) classification system for cardiotocography plus ST analysis and corresponding management guidelines……………………………………………………………………….6

[5. Supplementary tables (S2–S16) 7](#_Toc136420201)

[6. Appendix S3. START trial statistical analysis plan 19](#_Toc136420202)

[7. Appendix S4. STan protocol adherence 35](#_Toc136420203)

[8. References 37](#_Toc136420204)

### **Appendix S1** Summary of Data Safety Monitoring Committee (DSMC)

An independent DSMC committee was established for this trial, and were notified of any safety outcome defined in the DSMC Charter, and included maternal death, maternal intensive care unit (ICU) admission, intrapartum fetal or neonatal death, unplanned neonatal intensive care unit (NICU) admission. The Committee were also notified in the event of severe adverse events (SAE) which included umbilical cord arterial pH ≤ 7.05 and 5-minute APGAR of 5 or less.

***DSMC Members***

**Chair: Associate Professor Diogo Ayres-de-Campos**Associate Professor, University of Lisbon; Chair, Department of Obstetrics, Santa Maria Hospital, Lisbon, Portugal.

Email: dayresdecampos@gmail.com

Discipline: Obstetrics

**Dr Scott Morris**Neonatal and Perinatal Medicine Specialist, Flinders Medical Centre, Adelaide, Australia.

Email: scott.morris@sa.edu.au

Discipline: Neonatology

**Dr Katherine Lee**Senior biostatistician, Murdoch Children’s Research Institute, Melbourne, Australia.

Email: katherine.lee@mcri.edu.au

Discipline: Biostatistics

### **Table S1** Clinical indications for continuous electronic fetal monitoring (CEFM) as per Royal Australian and New Zealand College of Obstetricians and Gynaecologists (RANZCOG) clinical guideline^1^

| **Antenatal and intrapartum factors that increase risk of fetal compromise.  Intrapartum CTG is recommended.** | |
| --- | --- |
| **Antenatal Risk Factors** | **Intrapartum Risk Factors** |
| Abnormal antenatal CTG | Induction of labour with prostaglandin/oxytocin |
| Abnormal Doppler umbilical artery velocimetry | Abnormal auscultation or CTG |
| Suspected or confirmed intrauterine growth restriction | Oxytocin augmentation |
| Oligohydraminos or polyhydramnios | Regional analgesia (e.g. epidural or spinal) |
| Prolonged pregnancy ≥ 42 weeks | Abnormal vaginal bleeding in labour |
| Multiple pregnancy | Maternal pyrexia ≥ 38°C |
| Breech presentation | Meconium or blood stained liquor |
| Antepartum haemorrhage | Absent liquor following amniotomy |
| Prolonged rupture of membranes (≥ 24 hours) | Prolonged first stage as defined by referral guidelines |
| Known fetal abnormality which requires monitoring | Prolonged second stage as defined by referral guidelines |
| Uterine scar (e.g. previous caesarean section) | Pre-term labour less than 37 completed weeks |
| Essential hypertension or pre-eclampsia | Tachysystole (more than five active labour contractions in ten minutes, without fetal heart abnormalities) |
| Diabetes where medication is indicated, or poorly controlled, or with fetal macrosomia | Uterine hypertonus (contractions lasting more than two minutes in duration or contractions occurring within 60 seconds of each other, without fetal heart rate abnormalities) |
| Other current or previous obstetric or medical conditions which constitute a significant risk of fetal compromise (e.g. cholestasis, isoimmunisation, substance abuse) | Uterine hyperstimulation (either tachysystole or uterine hypertonus with fetal heart rate abnormalities) |
| Fetal movements altered unless there has been demonstrated wellbeing and return to normal fetal movements |  |
| Morbid obesity (BMI ≥ 40) |  |
| Maternal age ≥ 42 |  |
| Abnormalities of maternal serum screening associated with an increased risk of poor perinatal outcomes (e.g. low PAPP-A <0.4MoM or low PlGF) |  |
| Abnormal placental cord insertion |  |
| Abnormal cerebroplacental ratio |  |
| **Conditions where an intrapartum CTG is not indicated when the condition occurs in isolation, but if multiple conditions are present, intrapartum CTG should be considered.** | |
| **Antenatal Risk Factors** | **Intrapartum Risk Factors** |
| Pregnancy gestation 41.0-41.6 weeks gestation | Maternal pyrexia ≥ 37.8°C and < 38°C |
| Gestational hypertension |  |
| Gestational diabetes mellitus without complicating factors |  |
| Obesity (BMI 30-40) |  |
| Maternal age ≥ 40 and < 42 years |  |
| AFI 5-8cm (or MVP 2-3cm) |  |

Abbreviations: CTG, cardiotocography; BMI, body mass index; PAPP-A, pregnancy-associated plasma protein A; PlGF, placental growth factor; AFI, amniotic fluid index; MVP, maximum vertical pocket.

### **Appendix S2** Description of study arms

***Certification and Training***

ST analysis (STan) of the fetal electrocardiogram was first introduced to Australia in November 2014 at the Women’s and Children’s Hospital (WCH) as standard of care for women requiring continuous electronic fetal monitoring (CEFM), alongside the existing option of cardiotocography (CTG) alone. Between July 2015 to January 2018 (commencement of START), CTG+STan was utilised in approximately 400 labours, approximately 3 patients per week. During START, CTG+STan was utilised in an average of 5.4 participants per week.

A comprehensive multipronged approach to extensive education was undertaken by the hospital. International experts were engaged for face-to-face initial teaching of all intrapartum clinicians as well as masterclasses to achieve a cohort of “superusers” to support clinical staff in the day to day use of STan. Two yearly mandatory sessions were facilitated by the research midwife (BS) and multiple choice examinations have been in place for all intrapartum clinicians at WCH.

Continuous electronic fetal monitoring was conducted by midwives and obstetricians who were trained in the use of CTG and STan monitoring (holding Fetal Surveillance Education Program (FSEP) CTG accreditation, as well as in-house, institutional accreditation for competency at STan use and interpretation.

***Treatment arm (CTG+STan)***

A STan-capable monitor (Neoventa S31; Neoventa Medical, Mölndal, Gothenberg, Sweden) was connected to a tocodynometer on a belt applied to the woman’s waist. If not already *in situ*, a fetal scalp electrode (FSE) was applied to the occiput of the fetal scalp, and STan monitoring commenced, interpreted and acted upon according to the STAN guidelines (Figure S1). ^2^ In addition, any CTG abnormalities were interpreted and acted upon according to the RANZCOG guideline.^1^

***Control arm (CTG only)***

A CTG monitor (Philips or Neoventa S31; determined by what monitor already in the delivery room) was activated. A belt with a tocodynometer was applied to the woman’s waist. External monitoring of the fetal heart rate commenced by a belt-mounted Doppler monitor around her waist or, if clinically indicated, a FSE was applied and monitoring commenced and interpreted according to the RANZCOG guideline.^1^

***Umbilical cord gas measurements***

The Radiometer ABL90 Flex Plus (Radiometer Medical, Brønshøj, Denmark) was utilsed for cord blood arterial pH, venous pH and base excess in the extracellular component (BE_ecf_).

1. **Figure S1** Fetal heart rate classification system for cardiotocography plus ST analysis and management guidelines^2^

**
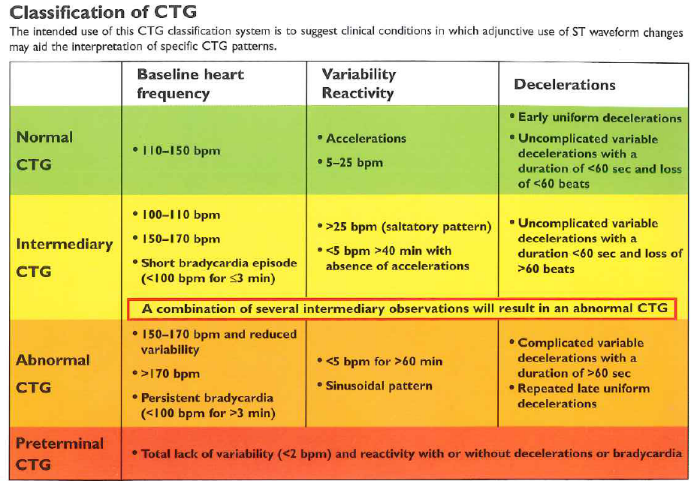
**

**
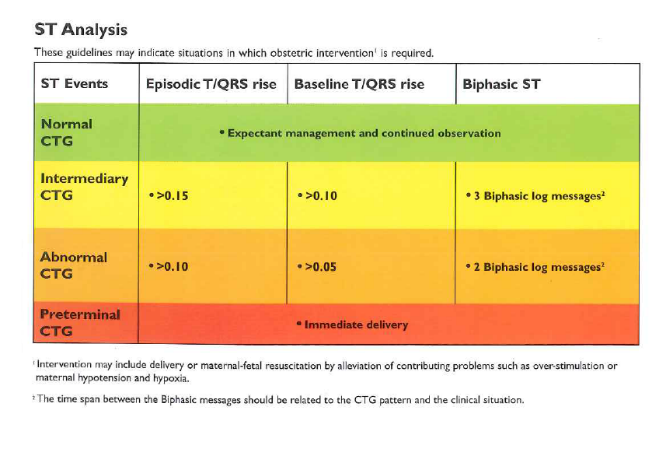
**

### **Supplementary tables**

**Table S2** Labor progress at time of consent

|  | **Randomised** | **Not randomised** | **All consents** | **% of total consents** |
| --- | --- | --- | --- | --- |
| Consent Prior to Labour | 263 | 234 | 497 | 37.11 |
| Consented in Early Labour | 405 | 105 | 510 | 38.08 |
| Consents after Epidural Analgesia | 295 | 31 | 326 | 24.35 |
| Consents in Established Labour* | 6 | 0 | 6 | 0.448 |
| **Total consents** | **969†** | **370** | **1339** | **100** |

* Ethics approval specified consenting only prior to, in early labour or after epidural analgesia. In the case of consents obtained in Established Labour, women were aware of the study and verbally consented prior to established labour, but written consent obtained when in established labour without epidural analgesia. †970 women were randomised overall, but only 969 had consented.

**Table S3** Reasons for consents not resulting in randomization

| **Reason** | **N (%)** |
| --- | --- |
| Trace too abnormal to initiate STan if randomised | 23 |
| Delivered too rapidly to facilitate the randomisation process | 10 |
| Consent not adequately communicated to midwifery staff | 7 |
| Woman wanted continued mobility and/or Transcutaneous Electrical Nerve Stimulation | 4 |
| CS indicated | 3* |
| Previous participation in START | 3 |
| Contraindicated after consent | 3† |
| Technical issue with FSE *in situ* and declining replacement | 1 |
| Changed mind about participating | 1 |
| Other | 3 |
| Unknown reason | 23 |
| **Total** | **81** |

*CS indicated for issues including hand presentation (1), breech (1), changed mind about induction of labour (1).
†Contraindicated after consent including fetal cardiac issue (1), CEFM not required (2).

**Table S4** Reasons for provisional consents* not resulting in randomisation

| **Reason** | **N (%)** |
| --- | --- |
| Condition of FSE clinically required and/or epidural analgesia not met | 233 |
| Condition/s met, but FSE applied during second stage of labour when STan cannot be initiated/delivering rapidly | 20 |
| Condition/s met but trace too abnormal to initiate STan if randomised | 13 |
| Condition/s met but unknown why not randomised | 8 |
| CS indicated before condition/s met | 7 |
| Condition/s met but previous participation in START | 3 |
| CS shortly after condition met | 2 |
| Change of mind after condition/s met | 2 |
| Condition/s met but technical issue with FSE | 1 |
| **Total** | **289** |

*Acknowledging the reluctance for women to consent due to FSE and/or mobility concerns, ‘provisional’ consent was offered when extra recruitment staff were available (from September 2020 to June 2021) to streamline the consenting process. Women would provide written informed consent, under the provision that they would only undergo randomisation only if a FSE was required for routine clinical care outside of the trial and/or mobility concerns were voided due to initiation of epidural analgesia. This increased consent rates, however compared to ‘full consents’ (i.e. not provisional on the clinical use of FSE and/or epidural analgesia), of which 91% resulted in enrolment, only 28% of women provisionally consenting to participate in the clinical trial were enrolled.

**Table S5** Reason for induction of labor and continuous fetal monitoring

|  | **CTG+STan  (N=482)** | **CTG  (N=485)** | **Total  (N=967)** |
| --- | --- | --- | --- |
|  |  |  |  |
| **Reason for induction of labour*** |  |  |  |
| Diabetes: no./total inductions (%) | 103/400 (21.4) | 100/384 (20.6) | 203/784 (21.0) |
| Post dates: no./total inductions (%) | 59/400 (12.2) | 50/384 (10.3) | 109/784 (11.3) |
| Decreased fetal movement:  no./total inductions (%) | 53/400 (11.0) | 52/384 (10.7) | 105/784 (10.9) |
| Macrosomia: no./total inductions (%) | 42/400 (8.7) | 52/384 (10.7) | 94/784 (9.7) |
| Other†: no./total inductions (%) | 223/400 (46.3) | 210/384 (43.3) | 433/784 (44.8) |
|  |  |  |  |
|  |  |  |  |
| **Reason for continuous fetal monitoring** |  |  |  |
| **Iatrogenic reasons**‡ |  |  |  |
| Epidural analgesia: no./total no. (%) | 409/482 (84.9) | 409/485 (84.3) | 818/967 (84.6) |
| Induction of labour: no./total no. (%) | 397/482 (82.4) | 382/485 (78.8) | 779/967 (80.6) |
| Oxytocin infusion: no./total no. (%) | 433/482 (89.8) | 418/485 (86.2) | 851/967 (88.0) |
| **Medical/obstetric reasons**§ |  |  |  |
| Diabetes: no./total no. (%) | 120/482 (24.9) | 127/485 (26.2) | 247/967 (25.5) |
| Hypertension or pre-eclampsia:  no./total no. (%) | 69/482 (14.3) | 67/485 (13.8) | 136/967 (14.1) |
| Maternal medical condition:   no./total no. (%) | 68/482 (14.1) | 63/485 (13.0) | 131/967 (13.5) |
| Other reason¶: no./total no. (%) | 235/482 (48.8) | 231/485 (47.6) | 466/967 (48.2) |
|  |  |  |  |
| * The four most common iatrogenic reasons for induction are reported. † Other reasons for induction include: intrauterine growth restriction: intrauterine growth restriction, iso-immunisation, fetal anomaly, antepartum haemorrhage, oligohydramnios, polyhydramnios, prolonged rupture of membranes, chorioamnionitis, poor obstetric history, cholestasis, hypertension, pre-eclampsia, advanced maternal age and maternal request/social. ‡ The three most common iatrogenic reasons for continuous fetal monitoring are reported. § The three most common medical/obstetric reasons for continuous fetal monitoring are reported. ¶ Other reasons for continuous fetal monitoring include: abnormal antenatal CTG, abnormal arterial Doppler, abnormal fetal heart rate, antepartum haemorrhage, breech, blood stained liquor, chorioamnionitis, iso-immunisation, intrauterine growth restriction, known fetal abnormality, maternal pyrexia, meconium stained liquor, oligohydramnios or polyhydramnios, post-term pregnancy ≥ 42 weeks, pre-term labour, prolonged active 1st stage of labour, prolonged rupture of membranes, per vaginal bleeding, reduced fetal movements, vaginal birth after caesarean or uterine scar. | | | |

**Table S6** Secondary per-protocol analysis* for primary outcome

| **Outcome** | **CTG+STan**  **(N=398)** | **CTG**  **(N=484)** | **Adjusted**† **Relative Risk  (95% CI)** | **P-value** |
| --- | --- | --- | --- | --- |
|  |  |  |  |  |
| **Emergency caesarean section** | 90 (22.6%) | 107 (22.1%) | 1.01 (0.80- 1.28) | 0.93 |
|  |  |  |  |  |

* Includes only those participants for whom fetal monitoring was performed according to study protocols.
† Relative Risk of emergency caesarean section (CTG+STan vs CTG) from log binomial regression model. Due to convergence issues, adjusted treatment effect estimate derived from log Poisson regression model with robust variance. Adjusted for parity (0 vs 1+), maternal BMI at baseline, private patient status, maternal age ≥35 years, previous CS delivery, higher risk delivery and induction of labour.

**Table S7** Secondary subgroup analyses for primary outcome

| **Outcome** | **CTG+STan no./**  **total no. (%)** | **CTG no./ total no. (%)** | | **Adjusted Relative Risk*** **(95% CI)** | **P-value** |
| --- | --- | --- | --- | --- | --- |
|  |  |  |  | |  |
| **Emergency Caesarean Section** | | | | | |
|  |  |  |  | |  |
| ***Effect modification by parity*** |  |  |  | | 0.84† |
| 0 | 88/289 (30.4%) | 86/290 (29.7%) | 1.03 (0.81, 1.32) | | 0.79 |
| 1 or more | 19/193 (9.8%) | 21/195 (10.8%) | 0.97 (0.58, 1.64) | | 0.92 |
|  |  |  |  | |  |
| ***Effect modification by BMI category*** |  |  |  | | 0.57† |
| <30 kg/m² | 53/294 (18.0%) | 64/313 (20.4%) | 0.92 (0.67-1.26) | | 0.59 |
| 30-34.9 kg/m² | 19/65 (29.2%) | 12/64 (18.8%) | 1.47 (0.80-2.72) | | 0.22 |
| 35-39.9 kg/m² | 17/65 (26.2%) | 12/48 (25.0%) | 0.96 (0.54-1.72) | | 0.89 |
| ≥40.0 kg/m² | 17/48 (35.4%) | 16/49 (32.7%) | 1.14 (0.68-1.91) | | 0.63 |
|  |  |  |  | |  |
| ***Effect modification by private patient status***‡ |  |  |  | | 0.70† |
| Public | 100/427 (23.4%) | 97/431 (22.5%) | 1.03 (0.82-1.30) | | 0.81 |
| Private | 7/55 (12.7%) | 10/54 (18.5%) | 0.86 (0.37-2.01) | | 0.74 |
|  |  |  |  | |  |
| ***Effect modification by maternal age ≥ 35 years*** |  |  |  | | 0.52† |
| Maternal age < 35 years | 83/382 (21.7%) | 84/374 (22.5%) | 0.98 (0.76-1.26) | | 0.89 |
| Maternal age ≥ 35 years | 24/100 (24.0%) | 23/111 (20.7%) | 1.17 (0.74-1.85) | | 0.51 |
|  |  |  |  | |  |
| ***Effect modification by previous CS delivery*** |  |  |  | | 0.47† |
| No | 100/463 (21.6%) | 95/455 (20.9%) | 1.04 (0.82-1.32) | | 0.74 |
| Yes | 7/19 (36.8%) | 12/30 (40.0%) | 0.78 (0.37-1.65) | | 0.52 |
|  |  |  |  | |  |
| ***Effect modification by higher risk delivery status***§ |  |  |  | | 0.12† |
| No | 59/292 (20.2%) | 69/295 (23.4%) | 0.88 (0.65-1.18) | | 0.39 |
| Yes | 48/190 (25.3%) | 38/190 (20.0%) | 1.26 (0.88-1.80) | | 0.20 |
|  |  |  |  | |  |
| ***Effect modification by induction of labour*** |  |  |  | | 0.69† |
| No (spontaneous or augmented) | 14/82 (17.1%) | 21/101 (20.8%) | 0.91 (0.50-1.66) | | 0.75 |
| Yes (induced) | 93/400 (23.3%) | 86/384 (22.4%) | 1.04 (0.81-1.32) | | 0.77 |
|  |  |  |  | |  |

* Relative Risk of emergency caesarean section (CTG+STan vs CTG) from log binomial regression model. Due to convergence issues, adjusted treatment effect estimates for effect modification by BMI category, private patient status, previous CS delivery, higher risk delivery and induction of labour are derived from log Poisson regression models with robust variance. Adjusted for parity (0 vs 1+), maternal BMI at baseline, private patient status, maternal age ≥35 years, previous CS delivery, higher risk delivery and induction of labour.

† P-value for interaction

‡ Public patient includes OBS/MFM/Shared Care/MGP, Midwives Clinic/Other. Private patient is private obstetrician (Women’s and Children’s Hospital).
§ Higher risk delivery is defined as one or more of: diabetes mellitus (Type 1/Type 2/GDM), hypertension (pre-existing/gestational), pre-eclampsia, IUGR, PPROM, Obstetric cholestasis

**Table S8** Sensitivity analysis for operative delivery in second stage of labor

| **Outcome** | **CTG+STan  (N=482 )** | **CTG  (N=485)** | **Adjusted Relative Risk* (95% CI)** | **P-value** |
| --- | --- | --- | --- | --- |
| **Operative delivery in Second Stage of Labour** | | | | |
| Operative (instrumental or CS) delivery in second stage of labour (%)† | 132 (27.4%) | 131 (27.0%) | 1.03 (0.84-1.25) | 0.73 |

* Relative Risk of outcome (CTG+STan vs CTG) from log binomial regression model, adjusted for parity (0 vs 1+), BMI at baseline and previous CS delivery.

†Outcome is defined only for the subset of women who reached the second stage of labour.

**Table S9** Secondary maternal outcomes: length of labor after randomization and length of second stage of labor

|  | **CTG+STan** | | **CTG** | |  |  |
| --- | --- | --- | --- | --- | --- | --- |
| **Outcome** | **No. events** | **Median duration (IQR)** | **No. events** | **Median duration (IQR)** | **Adjusted* difference in median duration (95% CI)** | **P-value** |
| **Length of labour after randomisation (hours)†** | 375 | 7.5  (4.3-11.2) | 378 | 7.3  (4.3-11.5) | -0.30  (-1.11-0.50) | 0.46 |
| **Length of second stage of labour (hours)‡** | 374 | 1.3  (0.4-2.3) | 378 | 1.3  (0.5-2.4) | -0.08  (-0.24-0.07) | 0.31 |

* Difference in median duration = estimated difference in median duration between treatment groups (CTG+STan vs CTG) from parametric survival model, adjusted for parity (0 vs 1+).
† Women who delivered by EmCS are considered censored.
‡ Outcome is defined only for the subset of women who reached the second stage of labour. Women who delivered by CS in the second stage of labour are considered censored. Note: a sensitivity analysis for this outcome was unnecessary as the length of second stage of labour was to be defined as 0 for participants who delivered prior to the second stage. Observations with time of 0 are ignored in survival modelling in Stata, such that treatment effect estimates for this outcome would remained unchanged with the inclusion of women who delivered prior to the second stage.

**Table S10** Maternal postrandomization characteristics

|  | **CTG+STan  (N=482 )** | **CTG (N=485)** | **Total**  **(n=967)** |
| --- | --- | --- | --- |
| **Maternal safety outcomes** | | | |
| Maternal death yes/total no. | 0 (0.0%) | 0 (0.0%) | 0 (0.0%) |
| Maternal ICU admission yes/total no. | 2 (0.4%) | 0 (0.0%) | 2 (0.2%) |
|  |  |  |  |
| **Complications of labour and delivery** | | | |
| Meconium stained liquor | 71 (14.7%) | 68 (14.0%) | 139 (14.4%) |
| Chorioamnionitis | 10 (2.1%) | 10 (2.1%) | 20 (2.1%) |
| Postpartum haemorrhage ≥500ml no./total no. (%)  *Severe (≥1000ml)* | 168 (34.9%)  50 (10.4%) | 193 (39.8%)  59 (12.2%) | 361 (37.3%)  109 (11.3%) |
| Shoulder dystocia  *Mild (no./total no. shoulder dystocia)*  *Moderate (no./total no. shoulder dystocia)*  *Severe(no./total no. shoulder dystocia)* | 19 (3.9%)  17/19 (89.5%)  2/19 ( 10.5%)  0/19 ( 0.0%) | 20 (4.1%)  17/20 (85.0 )  3/20 ( 15.0)  0/20 ( 0.0) | 39 (4.1%)  34/39 (87.2%)  5/39 ( 12.8%)  0/39 ( 0.0%) |
|  |  |  |  |
| **Maternal hospital length of stay (hours)** | | | |
| Mean (SD) | 76.9 (42.6) | 77.2 (40.7) | 77.1 (41.6) |
| Median (IQR) | 69.1 (47.5-93.5) | 68.8 (47.5-94.2) | 69.0 (47.5-93.6) |
|  | N=482 | N=484 | N=966 |
|  |  |  |  |
| **Maternal readmission after discharge (within 6 weeks of delivery)** | | | |
|  | 14 (2.9%) | 12 (2.5%) | 26 (2.7%) |
|  |  |  |  |

**Table S11** Neonatal postrandomization characteristics

|  | **CTG+STan**  **(N=482)** | **CTG**  **(N=485)** | **Total**  **(N=967)** |
| --- | --- | --- | --- |
| **Neonatal safety outcomes** |  |  |  |
| Infant death no./total no. (%) | 0 (0.0) | 0 (0.0) | 0 (0.0) |
| Intubation at delivery no./total no. (%) | 1^*†^ (0.2) | 0 (0.0) | 1 (0.1) |
| Neonatal seizures | 2^‡^ (0.4) | 0 (0.0) | 2 (0.2) |
| BRAINZ monitoring | 1^*^ (0.2) | 0 (0.0) | 1 (0.1) |
| Neonatal encephalopathy | 0 (0.0) | 0 (0.0) | 0 (0.0) |
| Use of infant cooling | 0 (0.0) | 1^§^ (0.2) | 1 (0.1) |
|  |  |  |  |
| **Major congenital malformation no./total no. (%)** | 4 (0.8) | 3 (0.6) | 7 (0.7) |
|  |  |  |  |
| **Fetal blood sampling** |  |  |  |
| Fetal scalp pH performed | 0 (0.0) | 1 (0.2) | 1 (0.1) |
| Fetal scalp lactate performed | 0 (0.0) | 0 (0.0) | 0 (0.0) |
|  |  |  |  |

* Did not receive STan as an adjunct to CTG.
† The case of intubation at birth was an expected outcome as the infant had congenital diaphragmatic hernia, diagnosed antenatally.
‡ Two seizures events were reported in neonates in the CTG+STan group but appear unrelated to labour outcomes; one seizure was unconfirmed and the other was readmitted at 4 weeks of age, after a hypoxic seizure at home with confirmed respiratory syncytial virus and rhinovirus.
§ Infant cooling was initiated in one infant in the CTG arm; observed for HIE, passively cooled to 33.9℃ initially, then warmed to normal.
¶ Other complications described included small lacerations, scabs, bruising, small scars, hair loss at FSE site and significant difficulty removing the FSE.

**Table S12** Postrandomization characteristics: fetal heart-rate-monitoring method and associated complications

|  | **CTG+STan**  **(n=482)** | **CTG**  **(n=485)** | **Total**  **(n=967)** |
| --- | --- | --- | --- |
|  |  |  |  |
| **External Doppler (continuous) only** | 30 (6.2%) | 130 (26.8%) | 160 (16.5%) |
|  |  |  |  |
| **FSE only** | 257 (53.3%) | 252 (52.0%) | 509 (52.6%) |
| ***Fetal scalp electrode in place for fetal heart rate monitoring prior to randomisation: most common reasons for application*** |  |  |  |
| Loss of contact n./total n. with FSE only | 136/257 (52.9%) | 127/252 (50.4%) | 263/509 (51.7%) |
| Abnormal trace n./total n. with internal monitoring only | 50/257 (19.5%) | 57/252 (22.6%) | 107/509 (21.0%) |
| Clinician preference n./total n. with internal monitoring only | 31/257 (12.1%) | 30/252 (11.9%) | 61/509 (12.0%) |
|  |  |  |  |
| **Both FSE and continuous Doppler during labour** | 194 (40.2%) | 103 (21.2%) | 297 (30.7%) |
|  |  |  |  |
| **Neither FSE nor continuous Doppler during labour** | 1 (0.2%) | 0 (0.0%) | 1 (0.1%) |
|  |  |  |  |
| **Complications from FSE use no./total no. with FSE** | 6/451 (1.3%) | 8/355 (2.3%) | 14/806 (1.7%) |
| Infection Bleeding Other^*^ | 1/451 (0.2%)  1/451 (0.2%)  4/451 (0.9%) | 0/355 (0.0%) 1/355 (0.3%)  7/355 (2.0%) | 1/806 (0.1%)  2/806 (0.2%)  11/806 (1.4%) |

*Other includes: small lacerations, scabs, bruising, scars, hair loss.

**Table S13** Continuous secondary neonatal outcome: 5-min Apgar score

|  | **CTG+STan** | | **CTG** | |  |  |
| --- | --- | --- | --- | --- | --- | --- |
| **Outcome** | **N** | **Mean (SD)** | **N** | **Mean (SD)** | **Adjusted*  Mean Difference (95% CI)** | **P-value** |
| **Infant Apgar score at 5 minutes** | 481 | 8.9 (0.5) | 485 | 8.9 (0.5) | 0.01 (-0.05-0.08) | 0.68 |

* Mean Difference from linear regression model, adjusted for parity (0 vs 1+).

**Table S14** Ordinal secondary neonatal outcome: level of neonatal admission and readmission

| **Outcome** | **CTG+STan**  **(N=482)** | **CTG**  **(N=485)** | **Adjusted† Odds Ratio  (95% CI)** | **P-value** |
| --- | --- | --- | --- | --- |
| **Neonatal admission to Neonatal Intensive Care Unit (NICU), Special Care Baby Unit (SCBU)  or admitted neonate on Postnatal Ward (Ward)** | | | | |
| ***Highest level of care*** | | | |  |
| None | 349 (72.4%) | 342 (70.5%) | 0.90 (0.68-1.19) | 0.46 |
| Ward | 28 (5.8%) | 28 (5.8%) |  |  |
| SCBU | 98 (20.3%) | 103 (21.2%) |  |  |
| NICU | 7 (1.5%) | 12 (2.5%) |  |  |
|  |  |  |  |  |
| **Neonatal re-admission to NICU, SCBU or Ward** | | | | |
| ***Highest level of care*** | | | |  |
| None | 444 (92.1%) | 435 (89.7%) | 0.74 (0.48-1.15) | 0.18 |
| Ward | 34 (7.1%) | 41 (8.5%) |  |  |
| SCBU | 4 (0.8%) | 8 (1.6%) |  |  |
| NICU | 0 (0.0%) | 1 (0.2%) |  |  |

* Odds Ratio of admission to higher level of care (vs lower levels of care) for CTG+STan vs CTG from ordinal logistic regression model, adjusted for parity (0 vs 1+).

**Table S15** Continuous secondary neonatal outcome: infant length of stay

|  | **CTG+STan** | | **CTG** | |  | |  | |  |
| --- | --- | --- | --- | --- | --- | --- | --- | --- | --- |
| **Outcome** | **N** | **Mean (SD)** | **N** | **Mean (SD)** | **Adjusted*  RM (95% CI)** | | **P-Value** | |  |
| **Infant length of stay** | | | | | | | | |  |
| No. hours between delivery and infant discharge | 482 | 61.2 (69.4) | 485 | 66.1 (99.2) | 0.93 (0.85, 1.02) | | 0.12 | |  |
| * RM = ratio of mean length of stay (CTG+STan vs CTG). Negative binomial regression model used due to overdispersion in the outcome, adjusted for parity (0 vs 1+). | | | | | |  | |  | |

**Table S16** DSMC severe adverse event reports*

|  | **CTG+STan**  **(N=10)** | **CTG**  **(N=15)** |
| --- | --- | --- |
| **Unplanned NICU admission** | **5†** | **11‡** |
| Not related to treatment | 4**§** | 4 |
| Unlikely related to treatment | 1¶ | - |
| Possibly related to treatment | - | - |
| **Maternal ICU admission** | **2** | **0** |
| Not related to treatment | 2 | **-** |
| Unlikely related to treatment | - | **-** |
| Possibly related to treatment | - | **-** |
| **Arterial cord pH ≤7.05** | **3** | **4‡** |
| Not related to treatment | - | - |
| Unlikely related to treatment | - | - |
| Possibly related to treatment | 3**ǁ** | 4 |
| **5 minute APGAR ≤5** | **1†** | **1** |
| Not related to treatment | - | - |
| Unlikely related to treatment | 1¶ | - |
| Possibly related to treatment | - | 1 |

* DSMC were blinded to treatment arm

† One report included unplanned NICU admission and 5 minute APGAR ≤ 5

‡ One report included unplanned NICU admission and arterial cord pH ≤7.05

§ Two of four did not receive STan monitoring during labour

¶ Did not receive STan monitoring during labour

ǁ STan monitoring ceased working in active second stage for one of the three cases of arterial cord pH ≤7.05

### **Appendix S3** START trial statistical analysis plan

Study Name: START

NHMRC Grant: 1129648

SAP Authors: Jennie Louise, Amy Salter and Bronni Simpson

(in discussion with Chris Wilkinson, Deborah Turnbull, Sabrina Kuah, Geoff Matthews, Andy McPhee and Edwin Chandraharan)

SAP Version: 10 (final version prior to analysis)

SAP Date: 07/12/2021

| **Abbreviation** | **Definition** |
| --- | --- |
| CRF | Case Report Form |
| CTG | Cardiotocography |
| SAP | Statistical Analysis Plan |
| STan | ST analysis: intrapartum fetal monitoring (cardiotocographic plus electrocardiographic) |
| START | STan intrapartum fetal monitoring (cardiotocographic plus electrocardiographic) compared with cardiotocographic (CTG) monitoring alone: an Australian randomised controlled trial |

PREFACE

This Statistical Analysis Plan (SAP) describes the planned analyses and reporting for the STan intrapartum fetal monitoring (cardiotocographic plus electrocardiographic) compared with cardiotocographic (CTG) monitoring alone: an Australian randomised controlled trial (START).

The following documents were reviewed in preparation of this SAP:

- START Trial Protocol (Version 27, 16 October 2018)
- START MIS Full Set of Requirements 15Nov2017

PURPOSE OF SAP

This SAP specifies the planned analyses to be conducted to support the completion of the primary paper for the START randomised controlled trial. It does not cover any secondary analyses, side studies, or follow-up studies.

AIMS AND HYPOTHESES

**Aims**

The primary objective of START is to determine the effect of STan intrapartum fetal monitoring (cardiotocographic plus electrocardiographic) compared to cardiotocographic (CTG) monitoring alone on the incidence of emergency caesarean section in women with pregnancies ≥ 36 weeks’ gestation, in whom caesarean delivery is not planned. Secondary aims are to determine the effect of STan monitoring, versus CTG monitoring alone, on a range of maternal and infant outcomes at delivery.

***Hypotheses***

The primary outcome variable is the incidence of emergency caesarean section, and the primary hypothesis is that the proportion of emergency caesarean sections will be lower in the CTG + STan monitoring group compared to the CTG monitoring group. The primary and secondary outcome variables are described in detail in Section 0.

**STUDY METHODS**

***Overall Study Design and Plan***

START is a randomised controlled trial of STan monitoring (CTG electronic fetal monitoring plus analysis of the ST segment of fetal electrocardiogram) versus CTG monitoring alone.

***Selection of Study Population***

Women 18 years or older, with a singleton fetus in cephalic presentation, who are able to give informed consent, are literate in English, and who do not meet any of the exclusion criteria, were eligible to participate in the trial. Criteria for exclusion were: pregnancy < 36 weeks’ gestation; having planned caesarean section delivery; placenta praevia or vasa praevia requiring caesarean delivery; contraindication for use of fetal scalp electrode; no clinical indication for continuous electronic fetal monitoring; or known fetal structural or functional cardiac conditions. Participation in START in a previous pregnancy was also an exclusion criterion.

***Method of Treatment Assignment and Randomisation***

After consent was obtained, eligible women were randomised to either the CTG + STan monitoring group (intervention group) or the CTG monitoring group (control group) in a 1:1 ratio, stratified for parity (0 vs 1+) according to a randomisation schedule prepared by a statistician not otherwise involved with the trial. Allocation was done via a telephone-based system provided by the NHMRC Clinical Trials Centre at the University of Sydney.

***Sample Size***

Original sample size calculations were based on data from a pilot study^3^, previous research, and rates of emergency caesarean section at the Women’s and Children’s Hospital, Adelaide. The proportion of emergency caesarean section deliveries in the CTG monitoring (control) group is expected to be approximately 17%, and the original sample size was based on providing 80% power (with two-sided $\alpha$=0.05) to detect a 5% absolute reduction in the STan + CTG group (i.e. from 17% to 12%). Allowing for 10% dropout after consent to participate, a further 22% attrition due to lack of clinical indication for fetal monitoring, and further 5% non-compliance in the CTG+STan group, it was estimated that 2588 women would need to consent, to allow for 1818 women to be randomised.

The number of women actually randomised to the study is approximately 970 (485 per group). With this number, the statistical power to detect the original difference of 5% is only ~ 56%; however there is still adequate power to detect differences which are only slightly larger: there is 80% power to detect an absolute reduction of 6.4% (proportion in CTG+STan group of 10.6%), and 70% power to detect an absolute reduction of 5.7% (proportion in CTG+STan group of 11.2%).

**SEQUENCE OF PLANNED ANALYSES**

An interim analysis was initially planned after recruitment of 800 women, for the purposes of reporting to the Data Safety Monitoring Committee. However, this was not conducted due to slow recruitment. As such, no interim analyses have been performed. No analyses will be performed until the final version of this SAP is complete, and has been approved by study investigators.

Analyses of primary and secondary outcomes described in this SAP will be performed once data collection and cleaning are complete. Analyses will be performed as specified in section 8 below; any deviations from the planned analyses will be clearly documented, along with the reasons for these, in a post-analysis version of the SAP. Following analysis, full results will be made available to the study investigators.

Any post-hoc, exploratory analyses which were not identified in this SAP, but are completed to support the planned study analyses listed in this SAP, will be clearly identified.

**GENERAL ISSUES FOR STATISTICAL ANALYSIS**

***Analysis Software***

All analyses will be performed using Stata v.15 (StataCorp, College Station, TX) or above.

***Analysis Approach***

The planned analyses will be performed using an intention-to-treat approach; randomised participants will be analysed according to the treatment they were randomised to receive. All participants with available outcome data will be included in the analysis. A secondary per-protocol analysis will be performed for the primary outcome only; including participants for whom monitoring (either CTG+STan or CTG alone) was performed according to study protocols.

***Methods for Withdrawals, Missing Data and Outliers***

Where a participant has withdrawn from the trial after randomisation, their data up to the point of withdrawal will be included in the analysis, provided that consent for data use has not also been withdrawn. Randomised women who did not receive the allocated intervention, but who have given consent to access medical records, will be included in the intention-to-treat population.

Missing data will not be imputed unless the proportion of missing data for the primary outcome exceeds 5%. Instead, complete case analysis will be used to analyse available data. If the proportion of missing data for the primary outcome is greater than 5%, multiple imputation will be used to create 100 complete datasets for analysis. Imputation will be performed separately by treatment group, using the Fully Conditional Specification (chained equations) method, and including outcomes, stratification variables, any covariates prespecified for adjustment in the analysis, and auxiliary baseline and post-randomisation variables as appropriate. In this case, analyses will be performed on both the imputed and raw data, with conclusions to be based on the analyses of imputed data. If imputation is performed, additional imputation models will also be performed to investigate the impact if primary outcome data are Missing Not At Random (MNAR); data will be imputed using pattern mixture models assuming that missing data have lower or higher risk of emergency caesarean section, in one or both groups.

Participants who experience an intrapartum fetal death will be excluded from analyses of neonatal outcomes, and will not have data imputed, since outcomes are undefined rather than missing in this case.

Outliers will be queried during data collection and the statistical analysis. Unless confirmed as a data entry error through verification of the CRF or other sources of data, or biologically implausible, outliers will not be excluded from the primary analysis.

***Data Transformations, Rare Events and Under- or Over-Dispersion***

No data transformations are planned. The statistical analyses described in Section 8 are based on assumptions about the distributions of the outcomes. These assumptions will be descriptively assessed prior to analysis, and if found to be invalid, appropriate data transformations may be investigated. In general, data transformations for continuous outcomes are not anticipated due to departures from normality, since the sample size is sufficient for the Central Limit Theorem to apply.^4^

Some binary outcomes may have insufficient events for the planned analysis to be sensible. In this case, the relevant outcomes will not be imputed, and comparisons between groups will be performed on the unimputed data using a Fisher’s Exact Test. If convergence problems are experienced with log binomial regression models, a log Poisson model with robust variance estimation will be used instead. In the case of further issues with convergence, some adjustment variables may be removed or modified.

If the assumptions of the Poisson model are violated for count outcomes (under- or over-dispersion, zero- inflation, or lack of zero counts), alternatives will be explored as appropriate, such as inclusion of a dispersion factor, negative binomial regression, zero-inflated or zero-truncated models.

***Covariates for Adjustment***

Both unadjusted and adjusted analyses will be performed for each outcome, with conclusions to be drawn from the adjusted analyses. All adjusted analyses will include parity (0 vs 1+), since this was a stratification variable used in randomisation, and this has been shown to induce correlation between treatment groups which leads to biased standard errors and reduction in statistical power if not adjusted for in the analysis.^5^ The following variables will also be included as covariates for some outcomes, as detailed in Section 8:

| Variable for Adjustment | Definition |
| --- | --- |
| Maternal BMI (kg/m^2^) | Continuous variable, measured at baseline |
| Private patient status | Binary variable (private vs public patient), derived from Antenatal Care Mode in baseline table:  Private Obstetrician (WCH) vs OBS/MFM/Shared Care/MGP, Midwives Clinic/Other |
| Maternal age ≥35 years | Binary variable: age < 35 years vs age 35+ years, defined from date of birth and date of randomisation in randomisation table. |
| Previous CS delivery | Binary variable (yes/no), from baseline table. |
| Higher risk delivery | Binary variable (yes/no), defined as one or more of the following:  - diabetes mellitus (Type 1/Type 2/GDM), as recorded in baseline table;  - hypertension (pre-existing/gestational), as recorded in baseline table;  - pre-eclampsia, as recorded in baseline table;  - IUGR, as recorded in baseline table;  - PPROM, as recorded in baseline table;  - Obstetric cholestasis, derived from baseline table (Other Complication, Specify) and maternal outcomes table (Reason for Induction) |
| Induction of labour | Binary outcome (yes/no), derived from Labour Type in maternal outcomes table: Induced vs Spontaneous+Augmented. |

If additional variables are included in analyses, these will be clearly identified in the post-analysis version of the SAP, along with the reason for inclusion.

***Planned Treatment by Covariate Interaction and Subgroup Analyses***

In addition to the primary analyses described in Section 8 below, secondary subgroup analyses will also be performed for the primary outcome only, to test for modification of the effect of treatment by the following baseline variables:

- Parity (0 vs 1+);
- BMI category: 18.5-24.9, 25.0-29.9, 30.0-34.9, 35.0-39.9, ≥40.0 kg/m^2^;
- Private patient status;
- Maternal age ≥35 years;
- Previous CS delivery;
- High-risk delivery;
- Induction of labour.

An interaction term between intervention and baseline variable will be included in the model, and separate estimates of the effect of intervention will be obtained for each level of the baseline factor, regardless of the statistical significance of the interaction term, since these comparisons are of interest *a priori*, and to allow comparison of results with those of other studies.

Any additional subgroup analyses or investigation of intervention-by-covariate interaction effects will be considered exploratory, and will be clearly identified as such.

***Multiple Comparisons and Multiplicity***

Multiple hypothesis tests are planned, due to the presence of multiple secondary outcomes, both unadjusted and adjusted analyses, and planned intervention-by- covariate interaction analyses, as well as analyses on both raw and imputed data if multiple imputation is performed.

No multiplicity adjustment will be made for the number of secondary outcomes analysed, or for planned intervention-by-covariate interaction analyses, since these analyses are of less importance, and less emphasis will be placed on the results.

Additionally, since conclusions will be drawn based on the results of adjusted analyses (or of adjusted analyses of imputed data if applicable), no multiple-comparisons adjustment will be made for the fact that both adjusted and unadjusted analyses will be performed (on both raw and imputed datasets) for some outcomes. Any statistically significant results (stipulated as two-sided p < 0.05) other than for the primary analysis of the primary outcome will be interpreted in the context of the inflated potential for Type I error due to multiple hypothesis tests.

***Intercurrent Events***

Some secondary outcomes described in Section 7 below are affected by intercurrent events, i.e., events occurring post-randomisation which affect the meaning or existence of the relevant outcome^6^:

- Length of second stage of labour;
- Operative delivery in second stage of labour.

These outcomes are undefined (do not exist) in women who do not reach the second stage of labour (due to delivery prior to second stage). The analysis of these outcomes will use a strategy analogous to the ‘while-treated’ strategy^6^, which analyses the outcome in participants while ‘on-treatment’, i.e. prior to development of the intercurrent event. In this case, the outcomes will be analysed in the subset of participants who reach the second stage of labour. This analysis approach assumes that there is no effect of treatment on the probability of reaching the second stage of labour.

As recommended by the ICH^6^, sensitivity analyses will be carried out for each outcome. For length of second stage of labour, a ‘composite variable’ strategy will be used, in which length of second stage of labour is defined as 0 for participants who delivered prior to the second stage. For operative delivery in second stage, those who delivered prior to the second stage of labour will be defined as ‘No’ for operative delivery in second stage of labour.

***Unblinding***

Sections 7 and 8 below describe all analyses to be performed to support the main paper for START. Some of these analyses involve outcomes which either require unblinding, or would provide strong indication of the identity of blinded treatment groups. The main analyses, involving the primary outcome and all secondary outcomes for which this is possible, will be performed by a statistician blinded to the identity of the treatment groups. Outcomes and analyses which require, or risk, unblinding, will be performed separately by another study investigator.

**DESCRIPTIVE STATISTICS**

***Flow Chart of Participants***

Information will be presented on:

- - Number of deliveries at Women’s and Children’s Hospital during the recruitment period;
  - Number of screened women not eligible as per exclusion criteria;
  - Number of women not screened;
  - Number of women screened and not approached and reason;
  - Number of approached and eligible women who did not consent, with most common reason/s;
  - Number of eligible women who consented to participate;
  - Labour progress at time of consent (prior to labour, early labour, established labour, post epidural); proportion of consents from each time resulting in randomisation;
  - Number of women randomised into each group;
  - Number of randomised women who withdrew, by treatment group;
  - Number of randomised women who received the allocated intervention, by treatment group;
  - Number of randomised women who did not receive allocated intervention, with reasons;
  - Number of women with data available for the primary outcome, by treatment group.

***Baseline Characteristics***

The following baseline characteristics will be presented, by treatment group and overall:

- Maternal age (yrs): mean (sd);
- Maternal BMI (kg/m2): mean (sd); plus proportions 30-34, 34-39 and 40+
- Private patient (yes/no): N(%);
- Parity (0 vs 1+) : N(%) ;
- Previous caesarean section: N(%);
- Diabetes (Yes/No): N(%);
- Hypertension (Yes/No): N(%);
- Pre-eclampsia (Yes/No): N(%)
- IUGR (Yes/No): N(%);
- PPROM (Yes/No): N(%);
- Obstetric cholestasis (Yes/No): N(%)
- Gestation at randomisation (weeks): median (IQR);
- Cervical dilation at randomisation (cm): mean (sd), median (IQR)
- Induction of labour (yes/no): N(%); plus proportions nulliparous (yes/no): N(%)
- Reason for induction of labour : N(%) of the four most common reasons for induction, with remaining reasons classified as ‘Other’: macrosomia, intrauterine growth restriction, decreased fetal movements, iso-immunisation, fetal anomaly, antepartum haemorrhage, oligohydramnios, polyhydramnios, prolonged rupture of membranes, chorioamnionitis, poor obstetric history, cholestasis, diabetes, hypertension, pre-eclampsia, post dates, maternal request/social, advanced maternal age, other;
- Oxytocin augmentation commenced before randomisation : N(%)
- Epidural analgesia before randomisation : N(%)
- Infant birth weight (g): mean (sd); plus proportions ≤2500 and ≥4000g
- Infant sex (male/female): N(%)
- Reason for continuous fetal monitoring :
  - Number of reasons for continuous monitoring (median, mean or categories as appropriate);
  - N (%) of the 3 most common iatrogenic reasons: epidural, induction of labour, oxytocin infusion;
  - N (%) of the 3 most common medical/obstetric reasons: abnormal antenatal CTG, abnormal arterial Doppler, abnormal fetal heart rate, antepartum haemorrhage, breech, blood stained liquor, chorioamnionitis, diabetes, hypertension or pre-eclampsia, iso-immunisation, intrauterine growth restriction, known fetal abnormality, maternal medical condition, maternal pyrexia, meconium stained liquor, oligohydramnios or polyhydramnios, post term pregnancy ≥ 42 weeks, pre-term labour, prolonged active 1^st^ stage of labour, prolonged rupture of membranes, per vaginal bleeding, reduced fetal movements, vaginal birth after caesarean or uterine scar

Continuous and count variables will be summarised using means and standard deviations, or medians and interquartile ranges as appropriate. Categorical variables will be presented as frequencies and percentages.

***Post-randomisation characteristics***

The following post-randomisation characteristics will be assessed descriptively by treatment group and overall:

*Mode of delivery*

As recorded in the Maternal Outcomes table:

- LCSC: N(%);
- Spontaneous vaginal: N(%);
- Instrumental delivery: forceps: N(%);
- Instrumental delivery: vacuum: N(%)

Derived from Maternal Outcomes table:

- Spontaneous vaginal with episiotomy: N(%);

*Cardio Monitoring Method*

Derived from Maternal Outcomes table:

- External only: N(%);
- Internal only: N (%);
  - If fetal scalp electrode in place for internal monitoring prior to randomisation, most common reasons for application, as N (%) of those with internal monitoring only;
- Both internal and external: N(%);
- Neither internal nor external: N(%)

*Maternal Safety Outcomes*

Outcomes recorded in the Maternal Outcomes table:

- Maternal ICU admission: N(%)
- Maternal death: N(%);
- Complications of Labour and Delivery:
  - Meconium stained liquor: N(%);
  - Chorioamnionitis: N(%);
  - Postpartum Haemorrhage ≥ 500mL: N(%);
  - Severe postpartum haemorrhage ≥ 1000mL: N(%);
  - Shoulder dystocia: N (%);
    - Severity of shoulder dystocia (mild/moderate/severe): N(%)
- Maternal hospital length of stay (discharge date – admission date);

Outcome recorded in the Maternal Postpartum table:

- Maternal readmission following discharge: N (%)

*Infant Safety Outcomes*

Outcomes recorded in the Maternal Outcomes table:

- Fetal scalp pH performed (yes/no): N (%)
- Fetal scalp lactate performed (yes/no): N(%)

Outcomes recorded in the Neonatal Outcomes table:

- Proportion of valid paired cord blood gases (both arterial and venous cord gases measured, and pH of arterial at least 0.03 lower than venous pH): N(%);
- Infant death (yes/no): N(%);
- Intubation at delivery (yes/no): N(%);
- Neonatal seizures (yes/no): N(%);
- BRAINZ Monitoring (yes/no): N(%);
- Neonatal Encephalopathy (yes/no): N(%);
- Hypoxic Ischaemic Encephalopathy (yes/no): N(%);
- Use of Infant cooling (yes/no): N(%)
- Major congenital malformation (yes/no): N(%);
- Complications from use of fetal scalp electrode: N(%)
  - Description of complications

*Study Quality Outcomes*

- Proportion of STan monitoring initiated correctly (i.e. on normal or intermediary trace) according to STan guidelines: N(%)
- STan recording quality:
  - Proportion of STan recording where STan was longer than 20 minutes in duration (N(%));
  - proportion of STan recordings where STan was not ceased within 20 minutes of delivery (N(%));
  - proportion of STan recordings where gaps in STan recording of ≥4 minutes occurred (i.e. where ST events may be missed) (N(%));
- Expedited delivery recommendations:
  - Proportion of STan recordings where there was a significant ST event in the second stage of labour (N(%));
  - Management of significant ST events in the second stage with regards to type and timing of delivery (i.e. was the infant delivered within STan guideline recommendations and if not, why not).

***Missing Data***

Missing data will be assessed descriptively by treatment group for each adjustment variable and subgroup analysis variable specified in Sections 6.5 and 6.6, each baseline and post-randomisation variable listed in Section 7 and each outcome variable specified in Section 0.

**STATISTICAL ANALYSES**

This section lists the primary outcome and secondary outcomes to be analysed, including their definition, the relevant section of the CRF, and the type of analysis to be performed, and estimate of treatment effect, for each.

***Primary Outcome***

| Outcome | Emergency Caesarean Section  Binary outcome, defined as delivery by caesarean section as recorded in the Maternal Outcomes table. |
| --- | --- |
| Analysis | Log binomial regression model |
| Adjustment | Parity, BMI, Private patient status, age ≥30, previous CS, high risk delivery, induction of labour. |
| Estimate | Relative Risk of emergency caesarean section (CTG+STan vs CTG alone) and 95% Confidence Interval. |

***Secondary Maternal Outcomes***

*Mode of Delivery*

| Outcome | Spontaneous vaginal birth (yes/no)  Binary outcome, defined as delivery by spontaneous vaginal birth versus caesarean section or instrumental vaginal delivery, as recorded in the Maternal Outcomes table. |
| --- | --- |
| Analysis | Log binomial regression model |
| Adjustment | Parity, BMI, previous CS |
| Estimate | Relative Risk of spontaneous vaginal delivery (CTG+STan vs CTG alone) and 95% Confidence Interval. |

*Reason for Caesarean Section*

| Outcome | Caesarean section delivery due to fetal distress (yes/no)  Binary outcome, defined based on reasons for caesarean section as recorded in the Maternal Outcomes table. Multiple reasons for caesarean section are possible; outcomes will be defined as caesarean section due to this reason, vs [caesarean section not due to this reason, or delivery not by caesarean section]. |
| --- | --- |
| Analysis | Log binomial regression model |
| Adjustment | Parity, BMI, previous CS |
| Estimate | Relative Risk of caesarean section due to each reason (CTG+STan vs CTG alone) and 95% Confidence Interval. |

*Reason for Instrumental delivery*

| Outcome | Instrumental delivery due to fetal distress (yes/no)  Binary outcome, defined based on reasons for instrumental delivery (forceps and vacuum-assisted) as recorded in the Maternal Outcomes table.  Multiple reasons for instrumental delivery are possible; outcomes will be defined as instrumental delivery due to this reason, vs [instrumental delivery not due to this reason, or delivery not by instrumental or caesarean section]. |
| --- | --- |
| Analysis | Log binomial regression model |
| Adjustment | Parity, BMI, previous CS |
| Estimate | Relative Risk of caesarean section due to each reason (CTG+STan vs CTG alone) and 95% Confidence Interval. |

*Oxytocin Augmentation*

| Outcome | Oxytocin augmentation commenced only after randomisation (yes/no)  Binary outcome, defined as Oxytocin Augmentation where Oxytocin Start Date/Time occurs after Randomisation Date/Time, as recorded in the Maternal Outcomes table. |
| --- | --- |
| Analysis | Log binomial regression model |
| Adjustment | Parity |
| Estimate | Relative Risk of caesarean section due to each reason (CTG+STan vs CTG alone) and 95% Confidence Interval. |

*Length of Labour after Randomisation*

| Outcome | Length of labour after randomisation  Continuous outcome, defined as number of hours between Randomisation Date/Time and Delivery Date/Time, as recorded in the Randomisation and Neonatal outcome tables. |
| --- | --- |
| Analysis | Linear regression model |
| Adjustment | Parity |
| Estimate | Difference in mean length of labour after randomisation (CTG+STan vs CTG alone) and 95% Confidence Interval. |

*Length of Second Stage of Labour*

| Outcome | Length of second stage of labour  Time-to-event outcome, derived from variables recorded in the Maternal Outcomes table.  The estimand for this outcome is duration of second stage of labour in women who did not deliver prior to the second stage; women who delivered by CS in the second stage of labour are considered censored. |
| --- | --- |
| Analysis | Parametric survival model |
| Adjustment | Parity |
| Estimate | Difference in median duration of labour (CTG+STan vs CTG alone) and 95% Confidence Interval. |

*Epidural analgesia*

| **Outcome** | Use of epidural after randomisation (yes/no).  Binary outcome, recorded in the Maternal Outcomes table. |
| --- | --- |
| **Analysis** | Log binomial regression model |
| **Adjustment** | Parity |
| **Estimate** | Relative Risk of use of epidural (CTG+STan vs CTG alone) and 95% Confidence Interval. |

*Operative Delivery in 2nd Stage of Labour*

| **Outcome** | Operative (Instrumental or CS) delivery in 2^nd^ stage of labour (yes/no)  Binary outcome, defined as delivery by instrumental vaginal delivery or caesarean in the second stage vs spontaneous vaginal delivery, as recorded in the Maternal Outcomes table. Outcome is defined only for the subset of women who reached the second stage of labour and is undefined for those who delivered prior to second stage. |
| --- | --- |
| **Analysis** | Log binomial regression model |
| **Adjustment** | Parity, BMI, previous CS |
| **Estimate** | Relative Risk of operative delivery in second stage of labour (CTG+STan vs CTG alone) and 95% Confidence Interval. |

*Composite Perinatal Outcome*

| **Outcome** | Composite of any of the following outcomes:   - - Infant death (intrapartum or neonatal)   - Apgar score ≤3 at 5 min   - Neonatal seizure   - Umbilical-artery blood pH ≤7.05 and base excess in extracellular fluid ≤-12 mmol/L   - Intubation for ventilation at delivery   - Presence of hypoxic ischaemic encephalopathy   Binary outcome, defined as any of the listed outcomes, as recorded on the Neonatal Outcomes table. |
| --- | --- |
| **Analysis** | Log binomial regression model |
| **Adjustment** | Parity |
| **Estimate** | Relative Risk of composite outcome (CTG+STan vs CTG alone) and 95% Confidence Interval. |

*Respiratory Distress at Delivery*

| **Outcome** | Respiratory distress at delivery  Binary outcome, as derived from the Neonatal Outcomes table. |
| --- | --- |
| **Analysis** | Log binomial regression model |
| **Adjustment** | Parity (0 vs 1+) |
| **Estimate** | Relative Risk of respiratory distress (CTG+STan vs CTG alone) and 95% Confidence Interval. |

*Neonatal Complications*

| **Outcome** | Specific neonatal complications:   - - Jaundice requiring phototherapy   - Meconium aspiration syndrome   - Use of antibiotics   - Confirmed infection   Binary outcomes, as recorded in the Neonatal Outcomes table. |
| --- | --- |
| **Analysis** | Log binomial regression model |
| **Adjustment** | Parity |
| **Estimate** | Relative Risk of individual neonatal complication (CTG+STan vs CTG alone) and 95% Confidence Interval. |

*Metabolic acidosis*

| **Outcome** | Metabolic acidosis:   - Umbilical-artery blood pH ≤ 7.05, and Base Excess ≤ -12.0 mmol/L; - Umbilical-artery blood pH ≤ 7.0, and Base Excess ≤ -12.0 mmol/L   Binary outcomes, derived from cord blood gas measures recorded in the Neonatal Outcomes table. |
| --- | --- |
| **Analysis** | Log Binomial regression model; no analysis for proportion of valid outcomes. |
| **Adjustment** | Parity |
| **Estimate** | Relative Risk of individual metabolic acidosis by cord gases (CTG+STan vs CTG alone) and 95% Confidence Interval. |

*Apgar Score at 5 minutes*

| **Outcome** | - Infant Apgar score at 5 minutes - Apgar score at 5 minutes ≤6, - Apgar score at 5 minutes ≤3   Apgar score: discrete outcome, to be treated as continuous, as recorded on the Neonatal Outcomes table.  Apgar scores ≤ 6 and ≤3 at 5 minutes: binary outcomes, derived from 5-minute Apgar score as recorded on the Neonatal outcomes table. |
| --- | --- |
| **Analysis** | Linear regression model (Apgar score at 5 minutes)  Log binomial regression model (Apgar score ≤ 6, Apgar score ≤3) |
| **Adjustment** | Parity |
| **Estimate** | Difference in mean Apgar score (CTG+STan vs CTG alone) and 95% Confidence Interval.  Relative Risk of Apgar score ≤6 / ≤ 3 at 5 minutes (CTG+STan vs CTG alone) and 95% Confidence Interval. |

*Neonatal Admission to NICU, SCBU or Qualified Neonate on Ward*

| **Outcome** | - Neonatal admission to NICU, SCBU or Qualified Neonate on Ward - Highest level of care (Neonatal Intensive Care Unit, Special Care Baby Unit, Qualified Neonate on Ward, None).   Outcomes derived from admission to NICU/SCBU/Qualified Neonate on Ward as recorded on Neonatal Outcomes table.  Neonatal admission to NICU/SCBU/Qualified Neonate: binary outcome defined as Yes to one or more of admission to NICU, admission to SCBU, qualified neonate on ward.  Highest level of care: defined as 0 (none of NICU/SCBU/Qualified neonate), 1 (Yes to Qualified Neonate, No to SCBU and NICU), 2 (Yes to SCBU and No to NICU), 3 (Yes to NICU). |
| --- | --- |
| **Analysis** | Neonatal admission to NICU/SCBU/Qualified Neonate: Log binomial regression model  Highest level of care: ordinal logistic regression model |
| **Adjustment** | Parity (0 vs 1+) |
| **Estimate** | Neonatal admission: Relative Risk of admission (CTG+STan vs CTG alone) and 95% Confidence Interval.  Highest level of care: Odds Ratio of admission to a higher level of care (vs lower levels of care) for CTG+STan vs CTG alone, and 95% confidence interval. |

*Neonatal re-admission to NICU, SCBU or Qualified Neonate on Ward*

| **Outcome** | - Neonatal re-admission to NICU, SCBU or Qualified Neonate on Ward - Highest level of care (Neonatal Intensive Care Unit, Special Care Baby Unit, Qualified Neonate on Ward, None).   Outcomes derived from admission to NICU/SCBU/Qualified Neonate on Ward as recorded on Neonatal Outcomes table.  Neonatal admission to NICU/SCBU/Qualified Neonate: binary outcome defined as Yes to one or more of re-admission to NICU, re-admission to SCBU, qualified neonate on ward.  Highest level of care: defined as 0 (none of NICU/SCBU/Qualified neonate), 1 (Yes to Qualified Neonate, No to SCBU and NICU), 2 (Yes to SCBU and No to NICU), 3 (Yes to NICU). |
| --- | --- |
| **Analysis** | Neonatal re-admission to NICU/SCBU/Qualified Neonate: Log binomial regression model  Highest level of care: ordinal logistic regression model |
| **Adjustment** | Parity (0 vs 1+) |
| **Estimate** | Neonatal re-admission: Relative Risk of admission (CTG+STan vs CTG alone) and 95% Confidence Interval.  Highest level of care: Odds Ratio of admission to a higher level of care (vs lower levels of care) for CTG+STan vs CTG alone, and 95% confidence interval. |

*Infant Length of Stay*

| **Outcome** | Infant length of stay.  Count outcome, defined as number of days between delivery date and date of infant discharge, as recorded in the Neonatal Outcomes section of the CRF. |
| --- | --- |
| **Analysis** | Log Poisson regression model |
| **Adjustment** | Parity (0 vs 1+) |
| **Estimate** | Ratio of mean length of stay (CTG+STan vs CTG alone) and 95% Confidence Interval. |

### **Appendix S4** ST analysis (Stan) protocol adherence

***Reason CTG+STan not received***

A total of 84 participants allocated to receive CTG+STan did not receive STan (CTG abnormal, could not initiate STan (23), delivered too rapidly to facilitate STan (15), fECG could not be obtained (15), declined FSE application after randomisation (9), obstetrically and/or medically inappropriate to place FSE and initiate STan (7), could not place FSE (6), other (9). Therefore, 398/482 (82%) of participants allocated to receive CTG+STan, had STan monitoring as an adjunct to CTG.

***Correct initiation***

In the trial institution, it was stipulated that the CTG be classified as normal or intermediary (by STan guidelines (see Figure S1)), prior to initiation of STan. Initiation occurred correctly (i.e. on a normal or intermediary trace) in 379/398 (95%) of cases.

***Gaps >4 minutes***

STan recordings were assessed for fetal ECG quality/consistency with regard to gaps ≥ 4 minutes, being that STan events may be missed.^2^ Gaps ≥ 4 minutes occurred in 135/398 (34%) traces, due to deliberate cessation of monitoring (38/135 (28%)) e.g. so that the woman could ambulate to void; due to fECG signal loss (69/135 (51%)); deliberate cessation and signal loss (27/135 (20%)); or other (due to FSE being replaced) (1/135 (0.7%)).

***Recordings <20 minutes***

The STan recording was for less than 20 minutes in one case (1/389 (0.3%)).

***Recordings ceased >20 minutes prior to delivery***

STan was ceased 20 minutes prior to delivery in 125/398 (32%) of cases. STan was deliberately disabled in 11/125 cases (8.8%) and STan ceased working in 6/125 (4.8%) of cases. In the majority of cases (108/125; 86.4%) STan was ceased >20 minutes prior to delivery due to transfer to the operating theatre for trial of forceps or CS.

***Management of STan events in the active second stage of labour***

Note: STan protocol adherence for management of STan events in the active second stage of labour was assessed by audit of the maternal clinical notes and the CTG+STan recording. Clinical factors, e.g. need for adequate analgesia for operative delivery, time to transfer to theatre and other situational influences were not considered in the reporting of the following.

A significant STan event is an event that warrants an obstetric intervention as per the guidelines (Figure S1). In the active second stage of labour, intervention should be operative delivery if spontaneous delivery is not anticipated within the next 5-10 minutes.^2^

Out of 472 women in the CTG+STan arm, 378/472 (80%) reached the second stage of labour (35 women had EmCS during the first stage of labour). There were STan events in the active second stage of labour in 72/378 (19%), of which (33/72; 46%) were not documented. The second stage event(s) were audited as significant in 46/72 (64%) cases, were not significant in 13/72 (18%) cases and occurred at the moment of delivery in 13/72 (18%) cases.

In the cases where the event was significant (operative delivery indicated, if not likely to spontaneously deliver within 5-10 minutes^2^), there were delays >10 minutes in 35/46 (76%) cases. Average delay to delivery after a significant ST event in the active second stage of labour was 46 minutes.

Of the 46 events audited as significant, the event(s) were part of the declared reason for operative delivery in 17/46 (37%), average delay to delivery was 40 minutes (range 16-116). A further 12 significant events were not documented resulting in delays to delivery (average delay of 40 minutes (range 12-119); 6 were misclassified as not significant (average delay of 106 minutes (range 20-182); 6 were not documented but delivered in <10 minutes. Documentation in case notes occurred in 3, which delivered in < 10 minutes. One case was misclassified as not significant, but delivered in <10 minutes.

### **References**

1. RANZCOG. Intrapartum Fetal Surveillance Clinical Guideline - Fourth edition. Royal Australian and New Zealand College of Obstetrics and Gynaecology 2019. https://ranzcog.edu.au/wp-content/uploads/2022/05/Intrapartum-Fetal-Surveillance.pdf [1 December 2022].
2. Amer-Wahlin I, Arulkumaran S, Hagberg H, Marsál K, Visser GH. Fetal electrocardiogram: ST waveform analysis in intrapartum surveillance. *Br J Obstet Gynaecol* 2007; **114** : 1191-1193.
3. Wilkinson C, Kuah S, Bryson K, Mayes M, Matthews G, Mol BW, Chandraharan E, McPhee A, Salter A, Symonds I, Turnbull D. A pilot randomised trial of STan fetal monitoring compared with CTG monitoring alone. In: abstracts of the 21st annual congress of the Perinatal Society of Australia and New Zealand (PSANZ), 2-5 April 2017, Canberra, Australia. *J Paediatr Child Health* 2017; **53** : 107.
4. Lumley T, Diehr P, Emerson S, Chen L. The Importance of the Normality Assumption in Large Public Health Data Sets. Ann Rev Public Health 2002; 23:151-169.
5. Kahan BC, Morris TP. Improper analysis of trials randomised using stratified blocks or minimisation. *Stat Med* 2012; **31** : 328-340.
6. ICH Expert Working Group. ICH Harmonised Guideline E9 (R1): Addendum on Estimands and Sensitivity Analysis in Clinical Trials. International Council for Harmonisation of Technical Requirements for Pharmaceuticals for Human Use. 2019. https://www.ema.europa.eu/en/documents/scientific-guideline/ich-e9-r1-addendum-estimands-sensitivity-analysis-clinical-trials-guideline-statistical-principles_en.pdf. [25 October 2022]
